# Supplementary material for: Engineering and exploiting synthetic allostery of NanoLuc luciferase
Source: Nat Commun. 2022 Feb 10;13:789. doi: 10.1038/s41467-022-28425-2 (PMC8831504; doi:10.1038/s41467-022-28425-2)
Supplement: Supplementary file 1 — Supplementary Information [file 41467_2022_28425_MOESM1_ESM.pdf]

Supplementary information

Engineering and exploiting synthetic allostery of NanoLuciferase

Zhong Guo, Rinky D Parakra, Ying Xiong, Wayne A. Johnston, Patricia Walden, Selvakumar Edwardraja, Shayli Varasteh Moradi, Jacobus P. J. Ungerer, Hui-wang Ai, Jonathan J Phillips and Kirill Alexandrov

Supplementary note 1: Analysis of cpNanoLuc tacrolimus biosensor specificity, activation rate and stability

To assess the suitability of the developed biosensors for diagnostic applications, we tested the effect of rapamycin and cyclosporine A on the performance of the tacrolimus biosensor. We titrated 1nM of tacrolimus biosensor either in buffer or in the presence of 200 molar excess of rapamycin (200nM) or 1000 molar excess of cyclosporine A (1μM). The data shown in the supplementary figure 1D shows that while neither rapamycin nor cyclosporin A led to activation of the tacrolimus biosensor both drugs interfered with its activation. This is not surprising as all biosensors share one common binding domain–FKBP between rapamycin and tacrolimus biosensors and Calcineurin A/ Calcineurin B complex between tacrolimus and cyclosporine A biosensors. Given the fact that even at 1000X drug excess the biosensor retained 7-10% of its dynamic range it is likely that one can use the developed biosensors to measure tacrolimus levels in the clinical samples containing both drugs. Further, it is possible that use of biosensor combination may allow even more accurate measurement of drug concentrations in such patients. Additional experiments are needed to ascertain this assumption.

To further characterize the performance of the developed Tacrolimus biosensor we tested the time dependence of its response to the ligand. We carried out the activity measurements following different incubation periods with the ligand. As shown in *supplementary figure* 2A the amplitude of response increased with increasing incubation time coming to saturation at 20 minutes. The full activity of the biosensor was determined to be about 20% compared to wt NanoLuc (Supplementary figure 2B).

We subsequently quantified the luminescent yield of the wild type NanoLuc, CaM-NanoLuc and the tacrolimus biosensor. As can be seen in supplementary figure 2B the CaM NanoLuc retained 70% of the luminescent yield of the parental enzyme while the tacrolimus biosensor retained 20% of the luminescent yield. This can be related to incomplete enzyme

reconstitution upon ligand binding or alternatively to changes in the Km values of NanoLuc resulting from mutagenesis. We observed that with increase of the furimazine concentration the signal increased linearly till it was too strong for the available instruments.

Next, we tested the stability of the tacrolimus biosensor under drying conditions. Supplementary figure 2C shows that the biosensor can be dried and rehydrated in functional form without the need of any optimization of the conditions. We subsequently tested the compatibility of the biosensor with lysed and whole blood. We observed that 50% hypotonically lysed blood significantly diminished the biosensor's signal (Supplementary figure 2D). This is not surprising as the emission wavelength of NanoLuc overlaps with the absorption wavelength of hemoglobin.

Emission absorption is a commonly encounter problem of Luciferase-based biosensors in biological systems. One of the ways of overcoming this is to employ a luminescent converter that absorbs light in the blue part of the spectrum and emits it at a higher wavelength in a phenomenon termed Bioluminescence Resonance Energy Transfer (BRET). We chose to test it using a green fluorescent protein that has matching excitation wavelength. In the context of the developed architecture introduction of such domain is not trivial as both N- and C-termini are occupied by large binding domains. Therefore, we decided to test this idea by using green fluorescent protein as a linker between the domains of the rapamycin biosensor.

Supplementary figure 3C shows the emission scan of such rapamycin biosensor in the absence and presence of rapamycin. It can be seen that the emission of the biosensor is shifted to the maximum of 510nm as expected and corresponds to the emission of EGFP. The results of titration of the red shifted biosensor with rapamycin are shown in supplementary figure 3D and demonstrate that despite the integration of the wavelength "extender" domain the biosensor faithfully produced enhanced light emission with the maximum at 510nm. The signal change of the biosensors in the presence of rapamycin could be fitted to a K_d of 0.5nM that demonstrates that despite introduction of the BRET-forming domain the function of the biosensor remained largely unchanged.

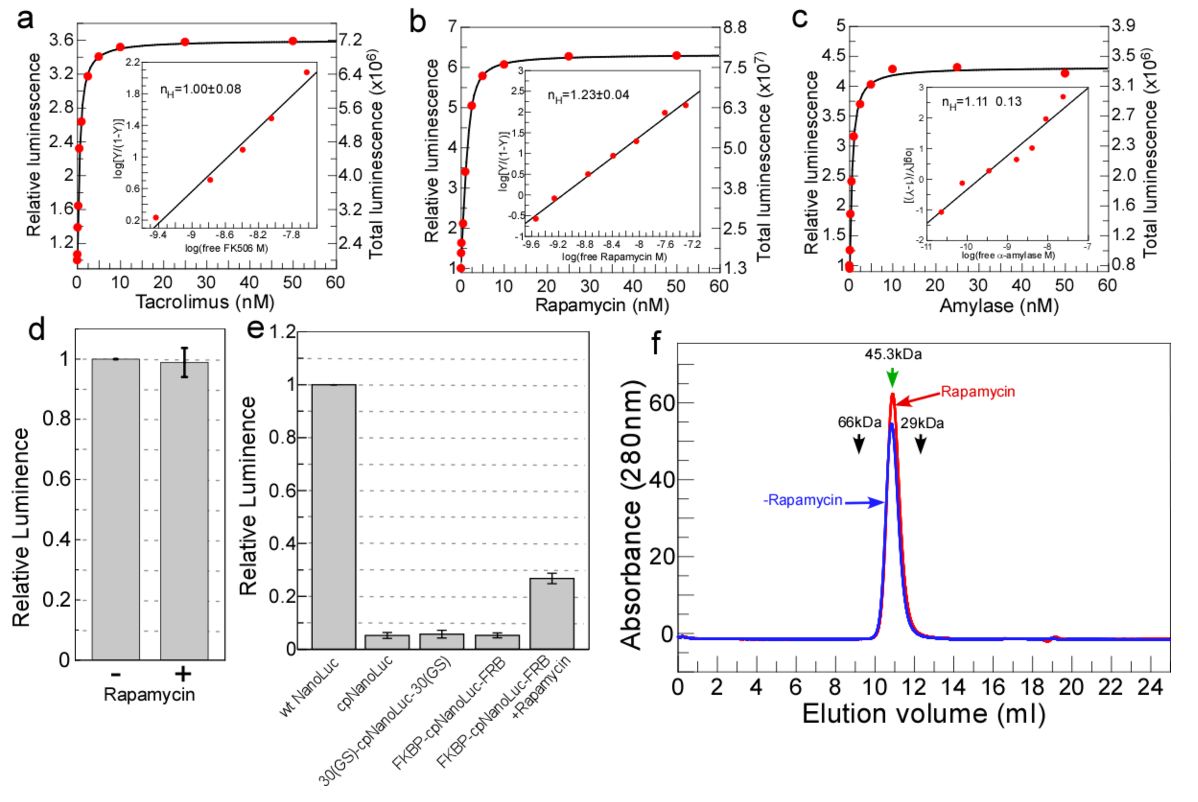

Supplementary figure S1 Analysis of cpNanoLuc biosensors of macrocyclic compounds and a-amylase. (A) Fit of the titration data for tacrolimus to a quadratic equation and the Hill coefficient (insert) (B) As in A but using rapamycin biosensor and rapamycin as titrant. (C) As in A but using the a-amylase biosensor and a-amylase as the titrant. The titrations were performed as described in Figure 1. (D) Activity analysis of 1nM solution of FRB-wtNanoLuc-FKBP fusion in 200μl buffer containing 20mM Tris pH7.4, 100mM NaCl and 0.1 μl NanoLuc substrate in the presence and absence of 50nM of Rapamycin. (E) Activity analysis of 1nM solutions of wild type NanoLuc, cpNanoLuc, cpNanoLuc with bearing 30 glycine, serine (GS) amino acid repeats on both N and C temini, FRB-cpNanoLuc-FKBP and FRB-wtNanoLuc-FKBP in the presence of 50nM rapamycin. The assay was performed as in (D), (F)Elution profile of Superdex 75 10/300 GL column loaded with 250μl of 1mg/ml solution cpNanoLuc of Rapamycin biosensor in the absence (blue graph) or presence (red graph) rapamycin. The black arrow heads indicate the positions of the gel filtration markers while green arrowhead indicates the calculated molecular weight of cpNanoLuc. In all plots the bars represent values of average of three independent measurements performed in the same experiment. The error bars denote positive and negative boundaries of the standard error of mean.

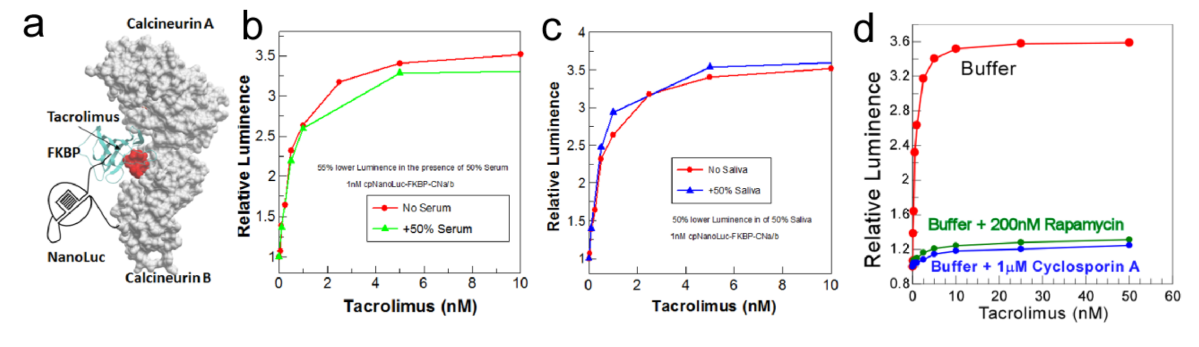

Supplementary figure 2 Structure and performance of cpNanoLuc tacrolimus biosensor (A) A model of a single component tacrolimus biosensor composed of circular permutated NanoLuc flanked with FKBP (displayed as ribbon) and a fusion of Calcineurin A and Calcineurin B proteins (displayed as a molecular surface). (B) Titration of 200μl solution of 1nM tacrolimus biosensor supplemented with 0.25μl furimazine stock solution in buffer containing 20mM Tris-HCl pH 7.2, 20mM NaCl with increasing concentrations of the drug in the presence or absence of 50% serum. The data was fitted to a K_d value of 0.4nM. (C) same as in (B) but in the presence or absence of 50% saliva. The data was fitted to a K_d of 0.4nM. (D) Analysis of the sensitivity of the tacrolimus biosensor to rapamycin. In the experiment 1nM solution of tacrolimus biosensor either alone of with 200nM rapamycin or 1mM cyclosporine A was titrated with increasing concentrations of tacrolimus.

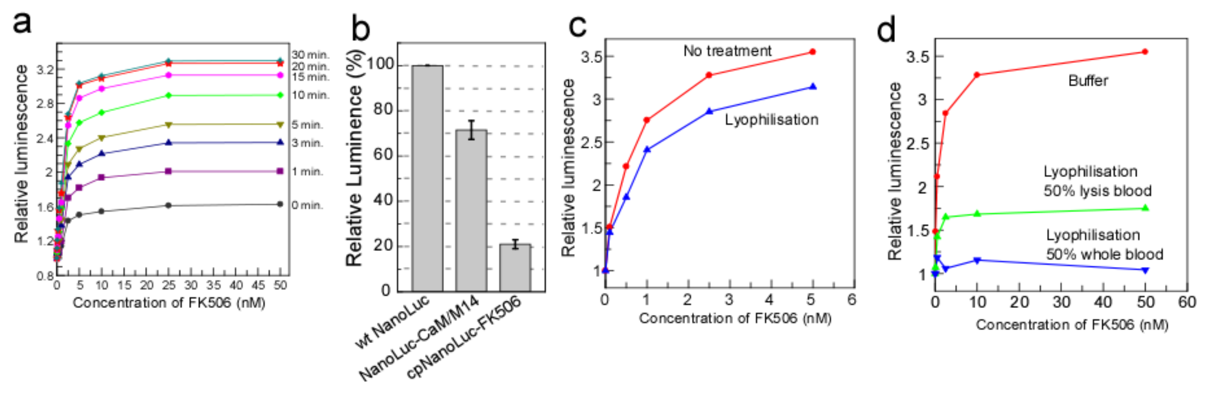

Supplementary figure 3 Assessing the performance of cpNanoLuc tacrolimus biosensor (A) Assessing the time dependence of biosensor activation. The stock reaction containing 1nM tacrolimus biosensor supplemented with 0.25μl/200μl furimazine stock solution were mixed with the indicated concentrations of tacrolimus and incubated for indicated periods of time in the 96 well plate. The luminescence of the samples was then measured and the data was plotted against the concentration of tacrolimus. (B) The comparison of the luminescence yield of 1nM solution of the wild type recombinant NanoLuc, CaM-NanoLuc in the presence

of 100nM of CaM-BP and NanoLuc-based tacrolimus biosensor in the presence of 50nM of tacrolimus. In the plot the bars represent values of average of three independent measurements performed in the same experiment. The error bars denote positive and negative boundaries of the standard error of mean. (C) Assessment of the ability of tacrolimus biosensor to withstand de- and rehydration. In the experiment 5μl of 40nM NanoLuc-based tacrolimus biosensor in assay buffer was dried in a 96 well plate and then re-suspended in 200μl of reaction buffer. The reactions were initiated by the addition of 0.25μl furimazine and indicated concentrations of tacrolimus and the samples were measured after 20 minutes incubation. In the control reactions the stock solution of the biosensors was diluted to the final concentration of 1nM with the reaction buffer, furimazine and the indicated concentrations of tacrolimus. (D) Assessment of tacrolimus biosensor performance in whole and lysed blood. The experiments were performed using as in C but in the presence of 50% of whole or hypotonically lysed blood.

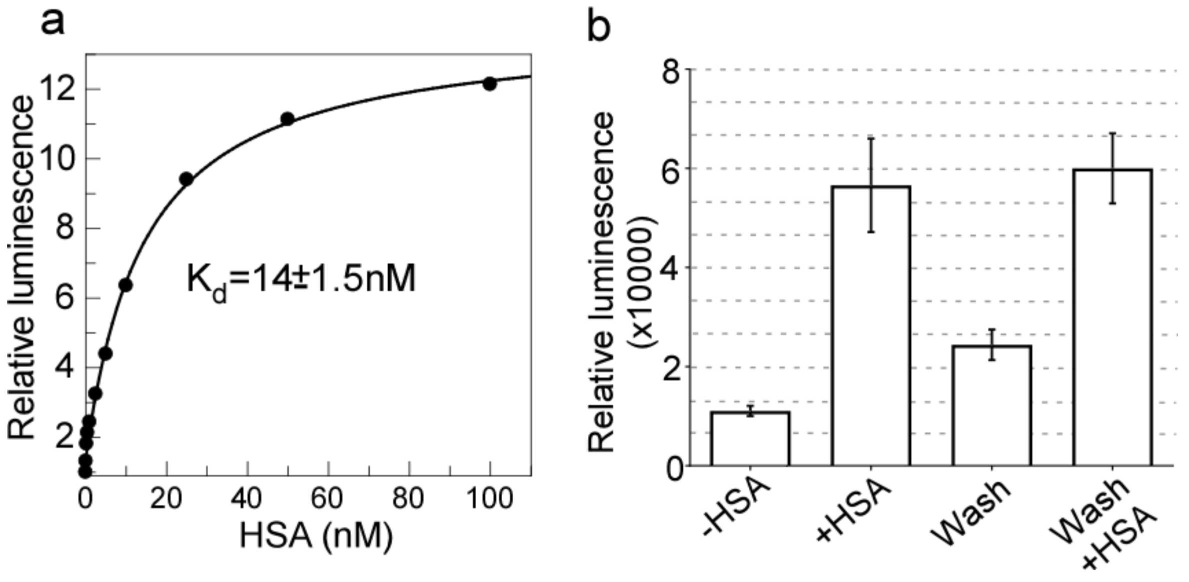

Supplementary figure 4. Construction of cpNanoLuc human serum albumin biosensor (HSA) and its interaction with the ligand. (A) Ligand titration experiment where 1nM solution of the biosensor was titrated with increasing concentrations of HSA. The relative luminescence was plotted against the concentration of the ligand and fitted to a K_d value of 14nM. (B) Activity analysis of cpNanoLuc HSA biosensor immobilized on the polystyrene plates and subjected to the repeated cycles of ligand binding and removal. In the plot the bars represent values of average of three independent measurements performed in the same experiment. The error bars denote positive and negative boundaries of the standard error of mean.

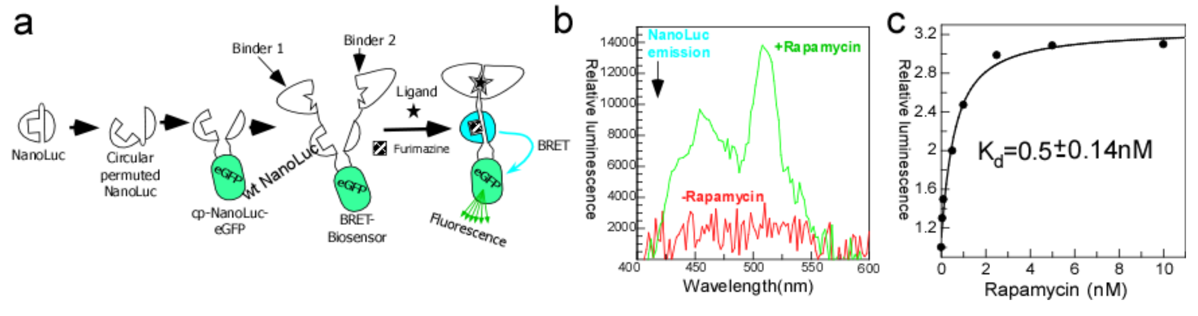

Supplementary figure 5 Construction of NanoLuc biosensors with red-shifted emission (A) The design sequence of converting wild type NanoLuc into a BRET sensor. The circular permutation of NanoLuc followed by the insertion of a fluorescent protein domain (in this case EGFP) between *de novo* created N- and C- terminus. The subsequent addition of the binding domains creates a biosensor that when activated by a ligand results in photon emission at 460nm that leads to fluorescent excitation of the fused EGFP that subsequently emits light with the emission maximum of 510nM. (B) A fluorescent scan of 1nM solution of rapamycin biosensor constructed as shown in (A). The red trace represents the emission of the biosensor in the absence of rapamycin while the green trace represents emission of the biosensor solution supplemented with 20nM of rapamycin. The maximum emission of NanoLuc is indicated by an arrow. (C) Titration of 1nM solution of rapamycin biosensor from A and B with increasing concentration of rapamycin. The luminescence over 480nm was recorded and the determined values were plotted against the concentrations of rapamycin. The data was fitted to the quadratic equation leading to a K_d value of 0.5nM which is very close to the values obtained for the parental biosensor.

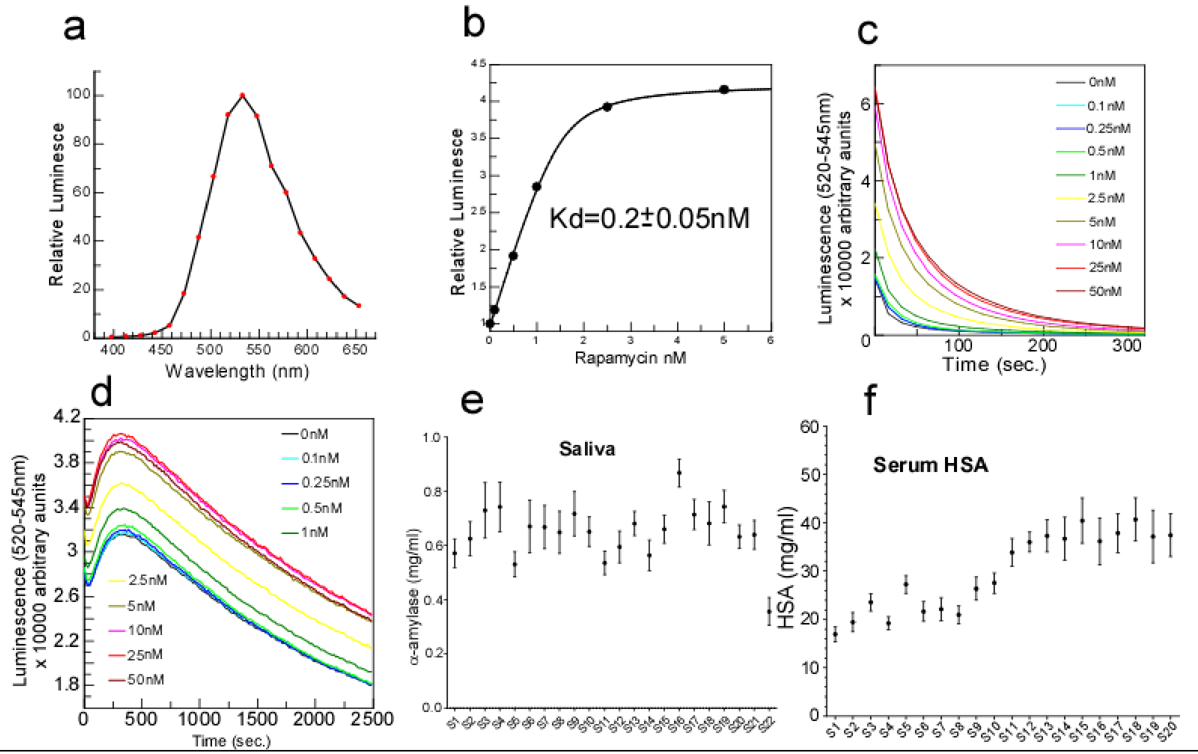

Supplementary figure 6 cpLumiLuc based rapamycin biosensors. (A) Emission scan of 1nM of cpLumiLuc Rapamycin biosensor in the presence of 100nM of Rapamycin and 30μM of 8pyDTZ. (B) Titration of 1nM of cpLumiLuc Rapamycin biosensor with the increasing concentrations of rapamycin. The data was fitted to a K_d value of 0.2nM. (C) Time-resolved changes in luminescence of a reaction mixture containing 1nM cpLumiLuc HSA sensor in buffer containing 20mM Tris-HCl pH 7.2, 100mM NaCl and 30mM 8pyDTZ as well as the indicated concentrations of HSA. (D) As in C but using optimized buffer containing 1mM CDTA, 0.5% Tergitol NP-40, 0.05% Antifoam 204, 150 mM KCl, 100 mM MES, pH 6.0, 1 mM DTT, and 35 mM thiourea. (E) Plot of the data of the a-amylase quantification assays based on cpLumiLuc performed in triplicates. (F) As in E but for serum HSA samples. The error bars denote positive and negative boundaries of the standard error of mean.

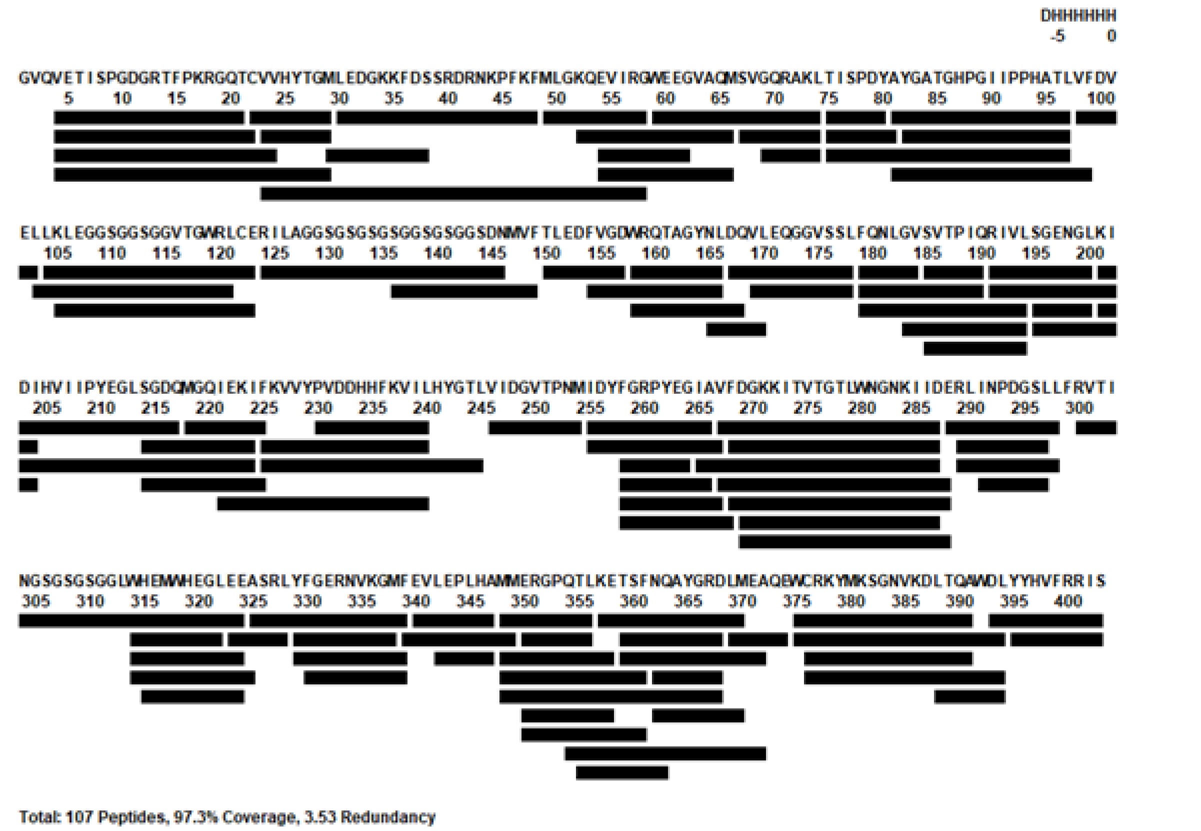

Supplementary figure 7. Mass spectral assignment (coverage map) of FKBP-cpNanoluc-FRB protein (rapamycin sensor). The black bars indicate the peptides, resulting from pepsin digest, that were identified by eight MS^E fragmentation experiments with different collision energy ramps, each with travelling wave ion mobility spectrometry (TWIMS) in N_2 gas enabled. Presented is the final pool of peptides that were monitored by HDX-MS and included for rapamycin dose-response analysis.

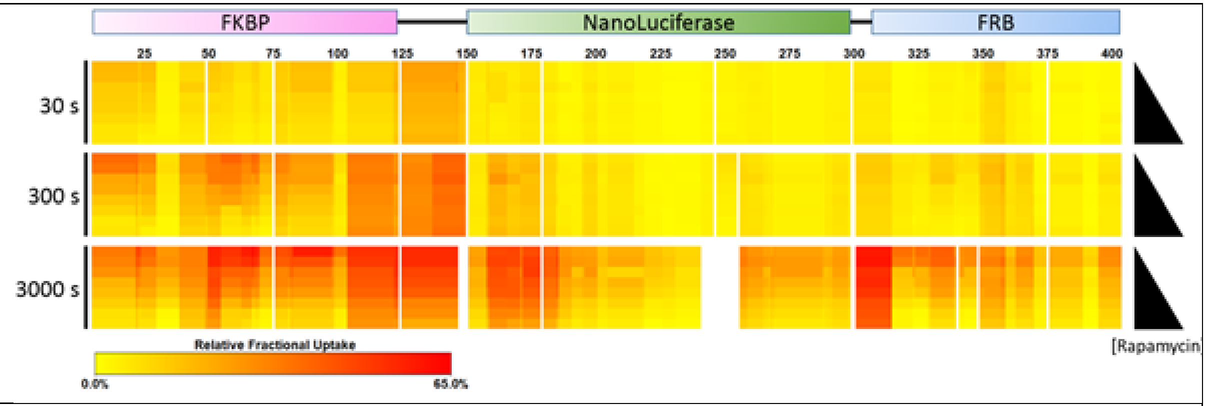

Supplementary figure 8. Heat maps for deuterium labelling at three mixing times (30, 300 and 3000 s) as a function of rapamycin concentration (0, 2, 4, 6, 8, 10, 15 and 20 μM) binding to 8 μM FKBP-cpNanoLuc-FRB. Protein domains and amino acid numbering are

shown above. The deuterium labelling is rendered at the single residue level as the mean of all overlapping peptides at each amino acid with linear weighting of the uptake across all exchangeable sites within those peptides. Created in DynamX 3.0 (Waters).

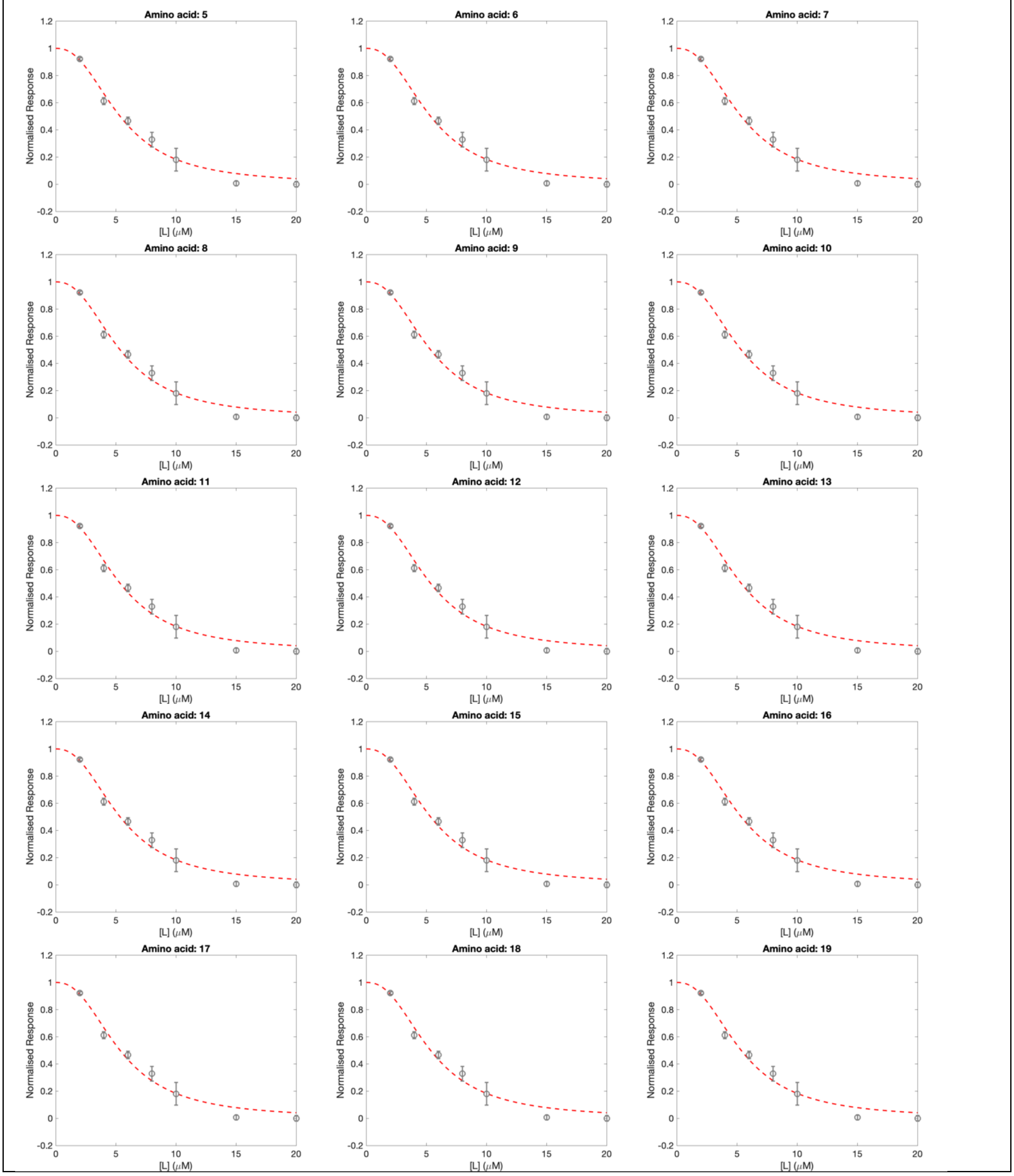

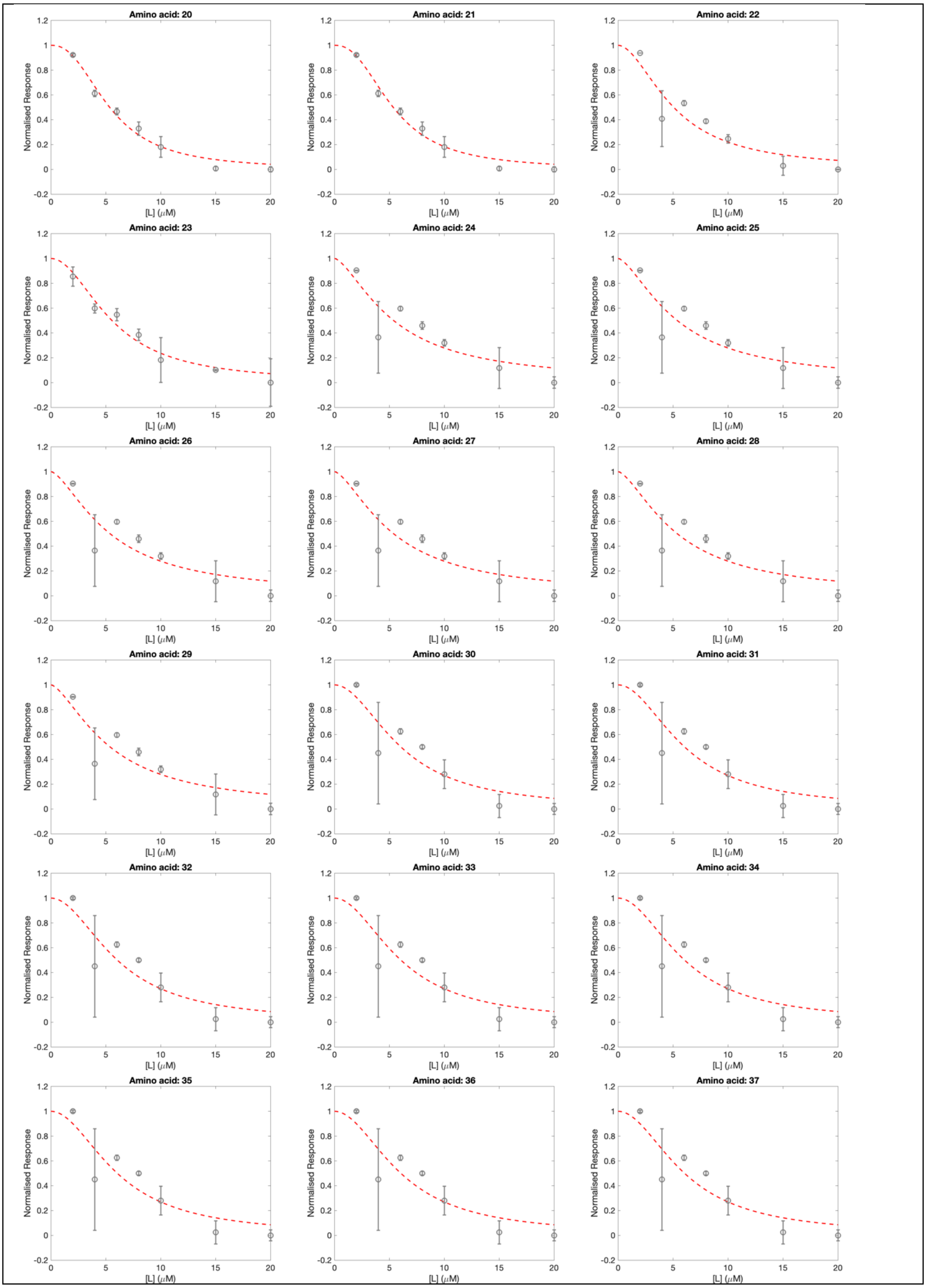
Amino acid: 20
Amino acid: 21
Amino acid: 22
Amino acid: 23
Amino acid: 24
Amino acid: 25
Amino acid: 26
Amino acid: 27
Amino acid: 28
Amino acid: 29
Amino acid: 30
Amino acid: 31
Amino acid: 32
Amino acid: 33
Amino acid: 34
Amino acid: 35
Amino acid: 36
Amino acid: 37
Normalised Response
[L] (μM)

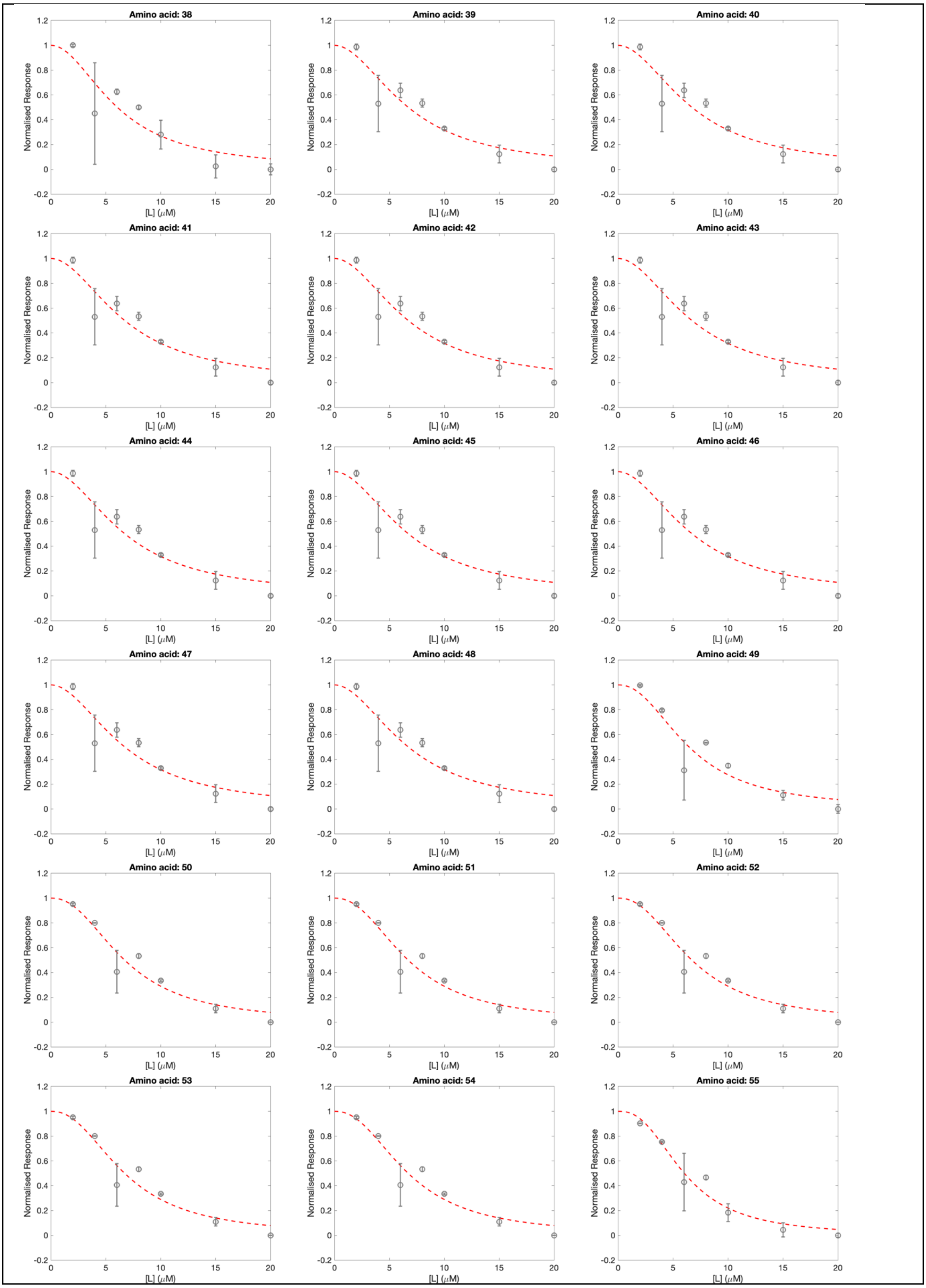
Amino acid: 38
Amino acid: 39
Amino acid: 40
Amino acid: 41
Amino acid: 42
Amino acid: 43
Amino acid: 44
Amino acid: 45
Amino acid: 46
Amino acid: 47
Amino acid: 48
Amino acid: 49
Amino acid: 50
Amino acid: 51
Amino acid: 52
Amino acid: 53
Amino acid: 54
Amino acid: 55
Normalised Response
[L] (μM)

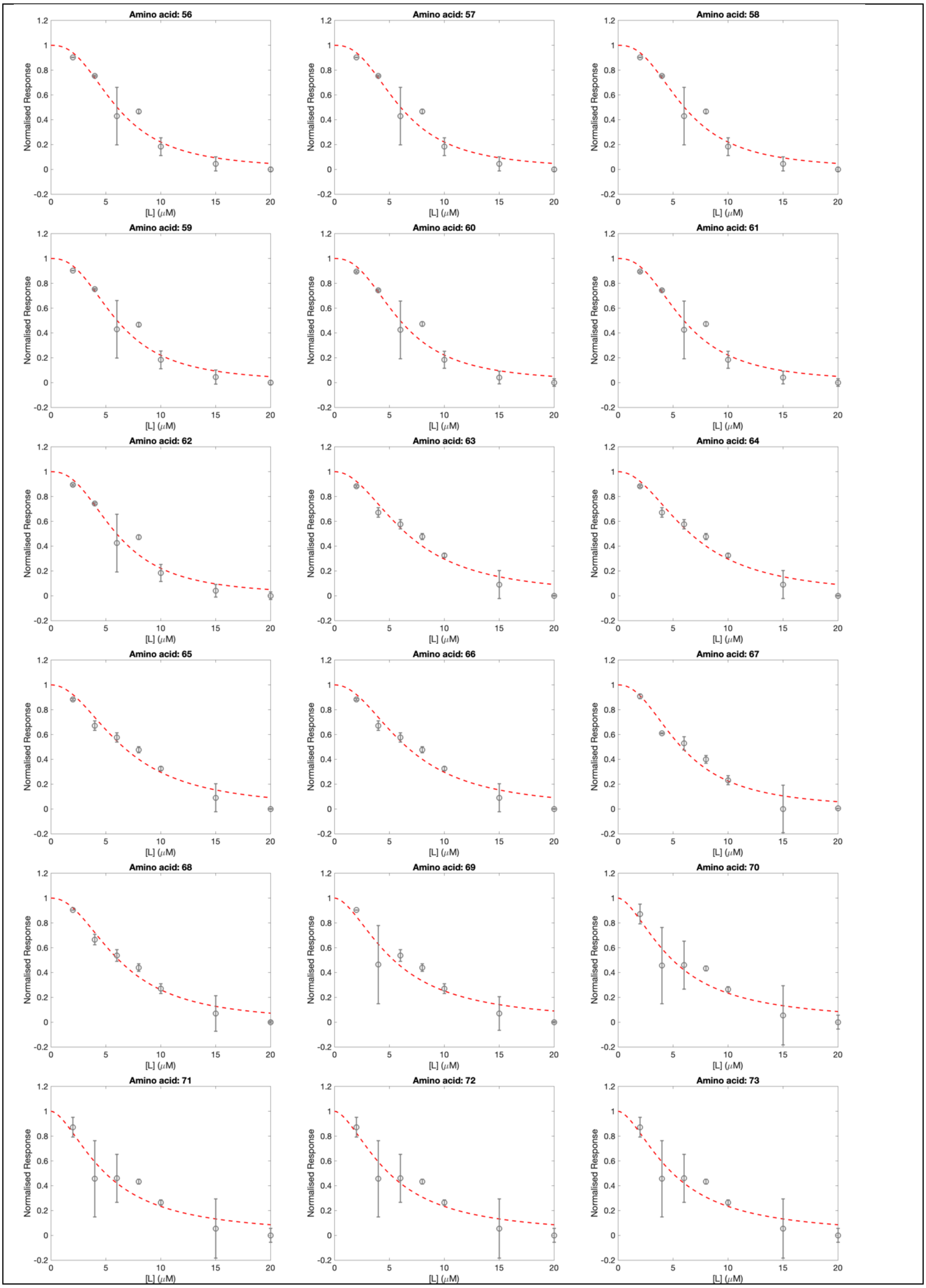
Amino acid: 56
Amino acid: 57
Amino acid: 58
Amino acid: 59
Amino acid: 60
Amino acid: 61
Amino acid: 62
Amino acid: 63
Amino acid: 64
Amino acid: 65
Amino acid: 66
Amino acid: 67
Amino acid: 68
Amino acid: 69
Amino acid: 70
Amino acid: 71
Amino acid: 72
Amino acid: 73
Normalised Response
[L] (μM)

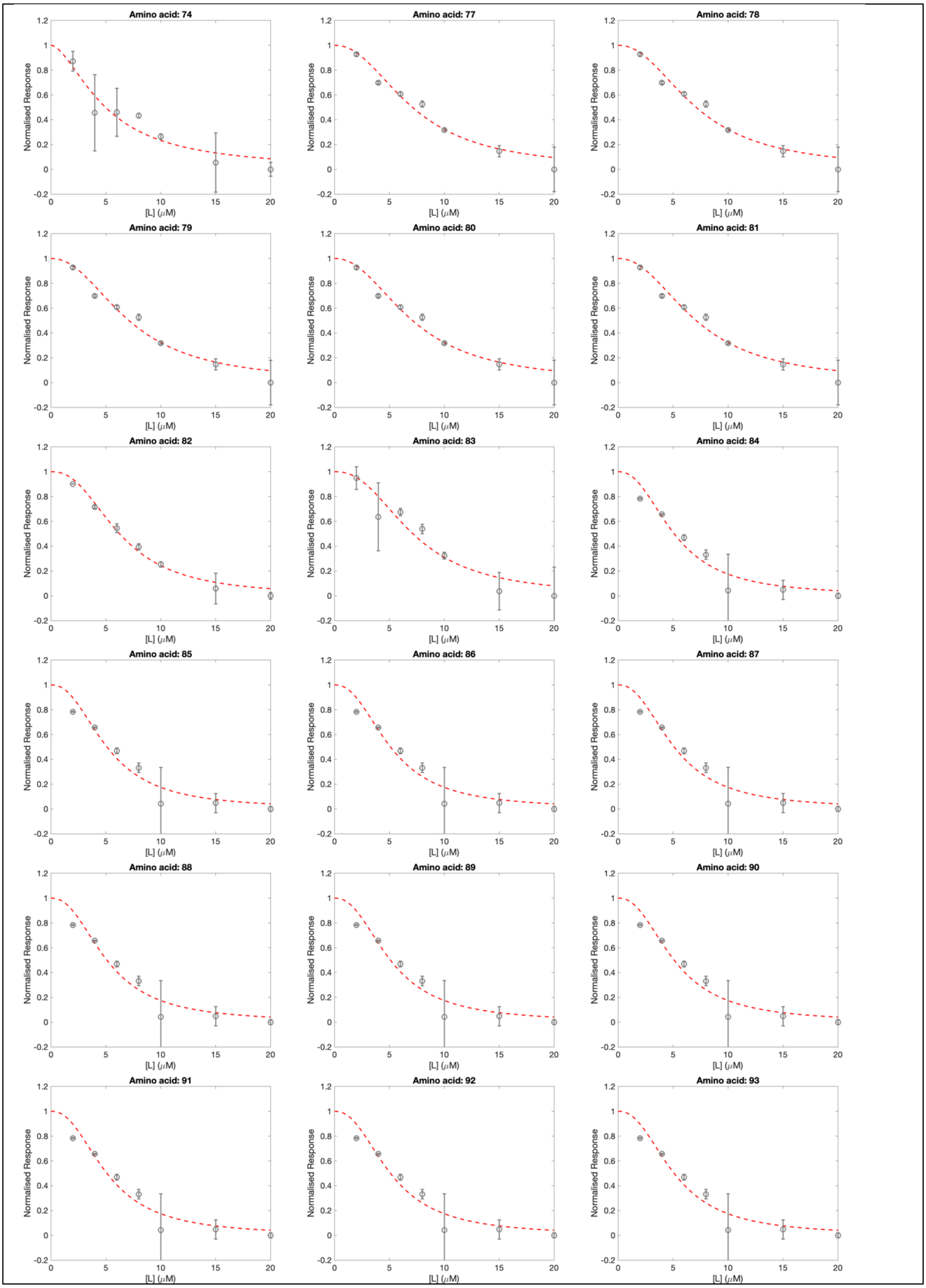
Amino acid: 74
Amino acid: 77
Amino acid: 78
Amino acid: 79
Amino acid: 80
Amino acid: 81
Amino acid: 82
Amino acid: 83
Amino acid: 84
Amino acid: 85
Amino acid: 86
Amino acid: 87
Amino acid: 88
Amino acid: 89
Amino acid: 90
Amino acid: 91
Amino acid: 92
Amino acid: 93
Normalised Response
[L] (μM)

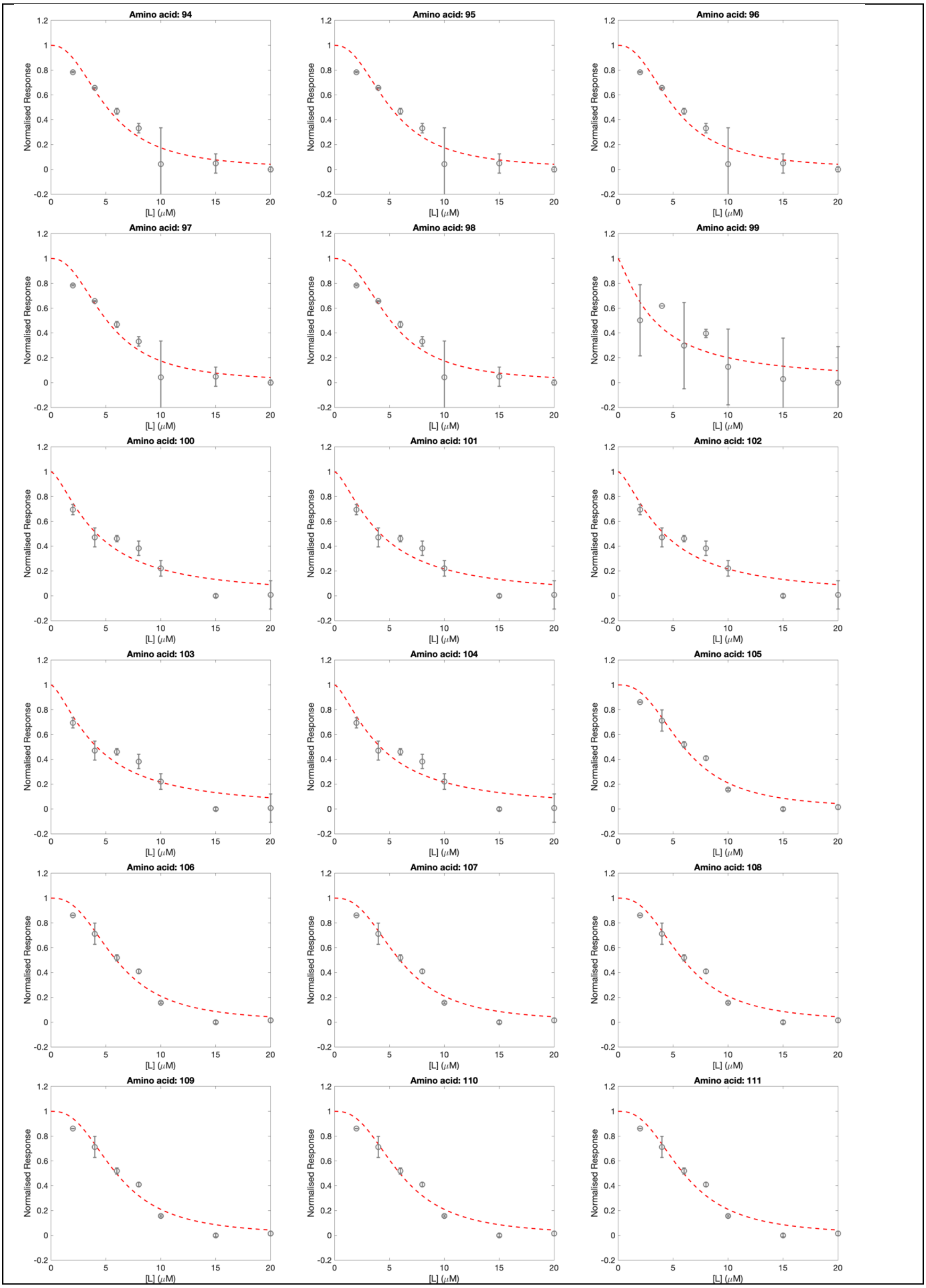
Amino acid: 94
Amino acid: 95
Amino acid: 96
Amino acid: 97
Amino acid: 98
Amino acid: 99
Amino acid: 100
Amino acid: 101
Amino acid: 102
Amino acid: 103
Amino acid: 104
Amino acid: 105
Amino acid: 106
Amino acid: 107
Amino acid: 108
Amino acid: 109
Amino acid: 110
Amino acid: 111
Normalised Response
[L] (μM)

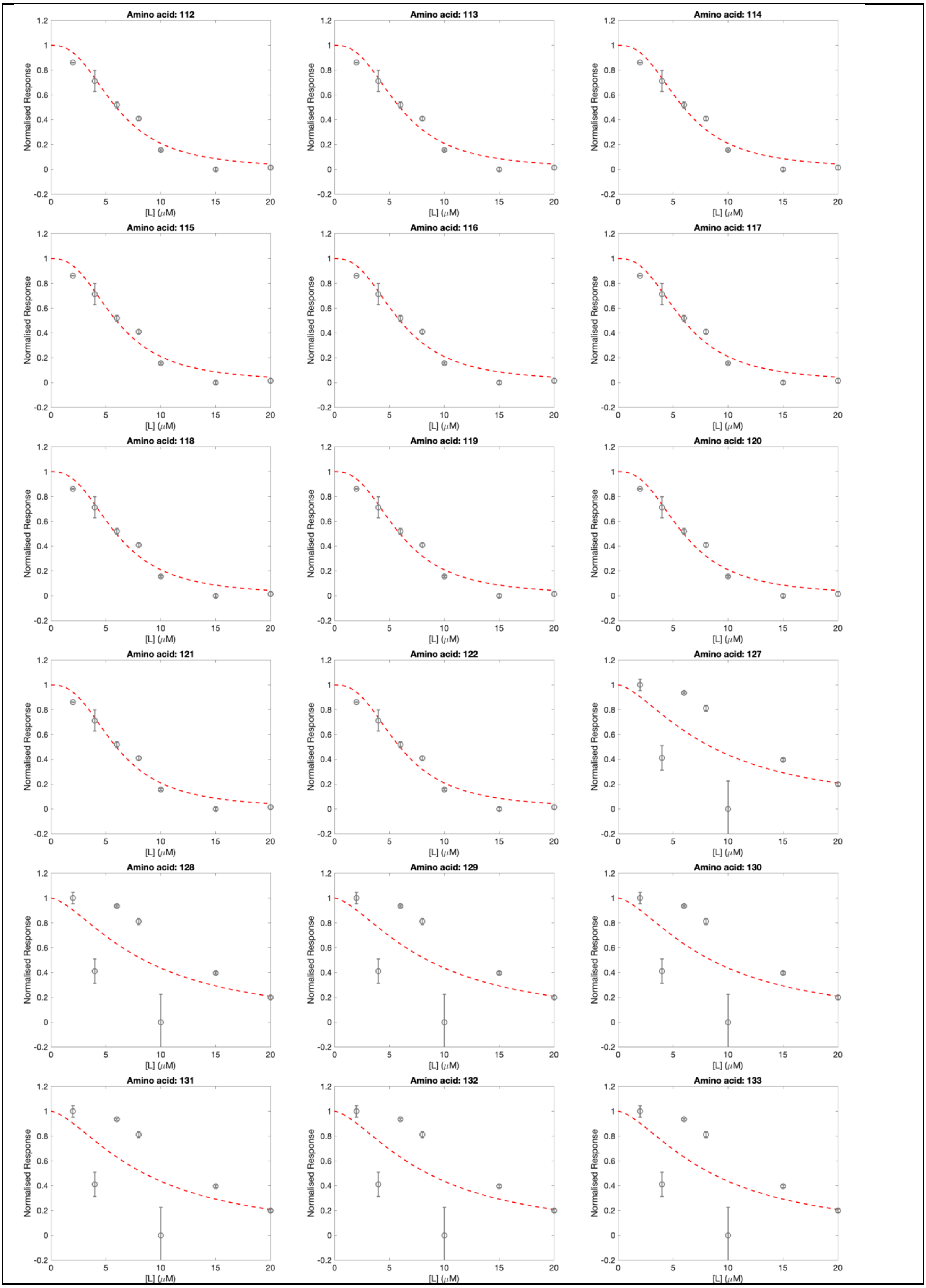
Amino acid: 112
Amino acid: 113
Amino acid: 114
Amino acid: 115
Amino acid: 116
Amino acid: 117
Amino acid: 118
Amino acid: 119
Amino acid: 120
Amino acid: 121
Amino acid: 122
Amino acid: 127
Amino acid: 128
Amino acid: 129
Amino acid: 130
Amino acid: 131
Amino acid: 132
Amino acid: 133
Normalised Response
[L] (μM)

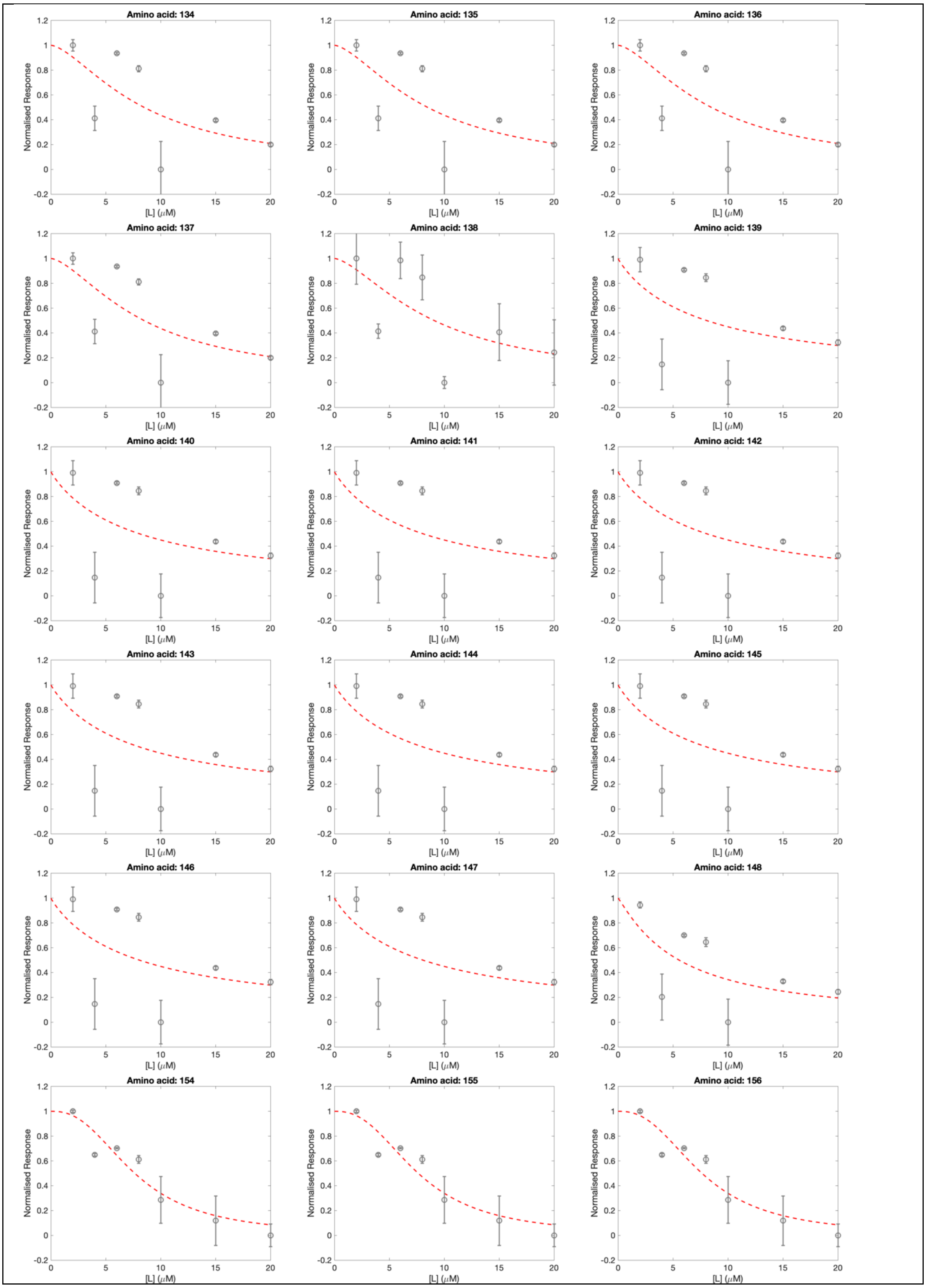
Amino acid: 134
Amino acid: 135
Amino acid: 136
Amino acid: 137
Amino acid: 138
Amino acid: 139
Amino acid: 140
Amino acid: 141
Amino acid: 142
Amino acid: 143
Amino acid: 144
Amino acid: 145
Amino acid: 146
Amino acid: 147
Amino acid: 148
Amino acid: 154
Amino acid: 155
Amino acid: 156
Normalised Response
[L] (μM)

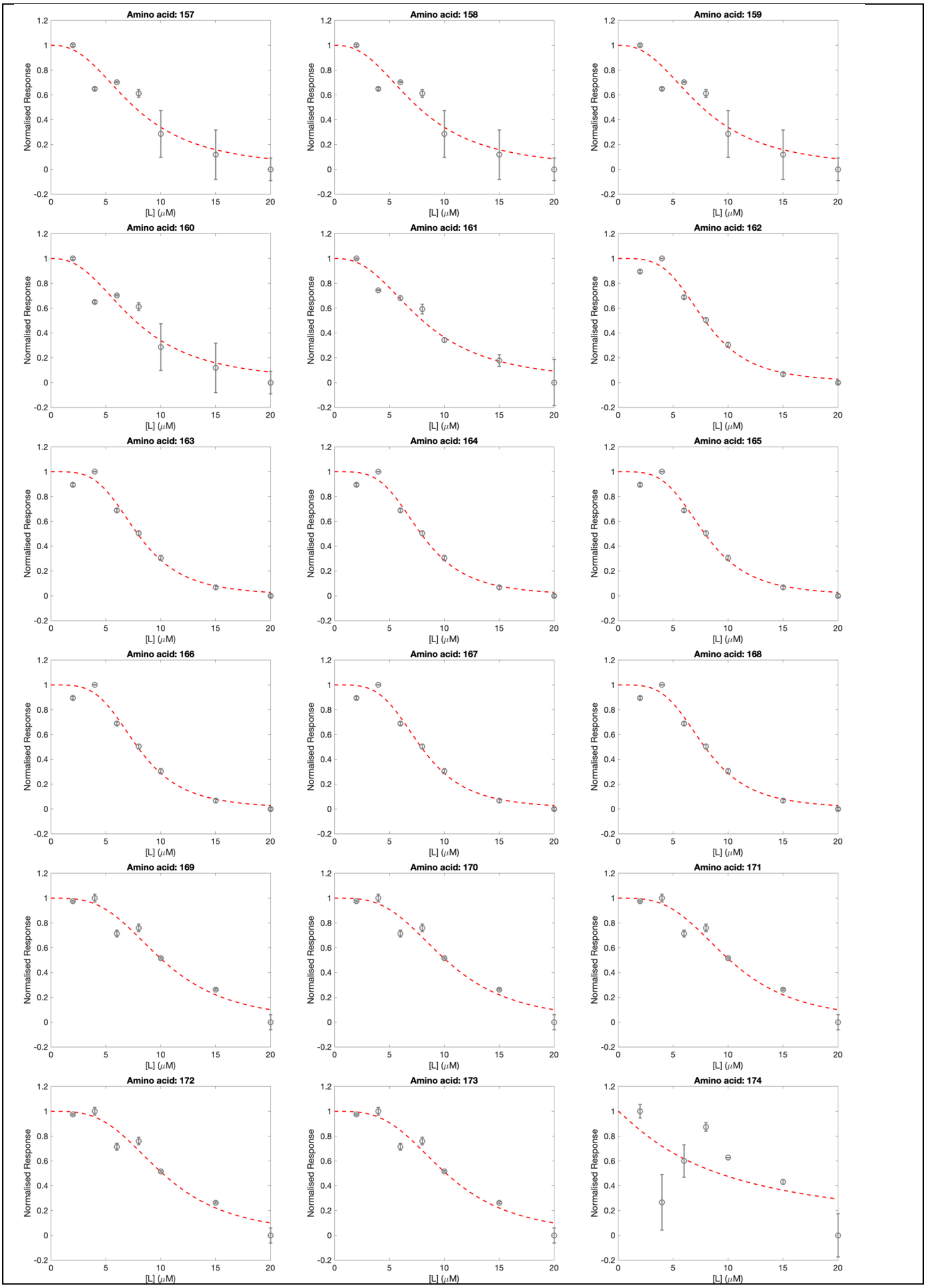
Amino acid: 157
Amino acid: 158
Amino acid: 159
Amino acid: 160
Amino acid: 161
Amino acid: 162
Amino acid: 163
Amino acid: 164
Amino acid: 165
Amino acid: 166
Amino acid: 167
Amino acid: 168
Amino acid: 169
Amino acid: 170
Amino acid: 171
Amino acid: 172
Amino acid: 173
Amino acid: 174
Normalised Response
[L] (μM)

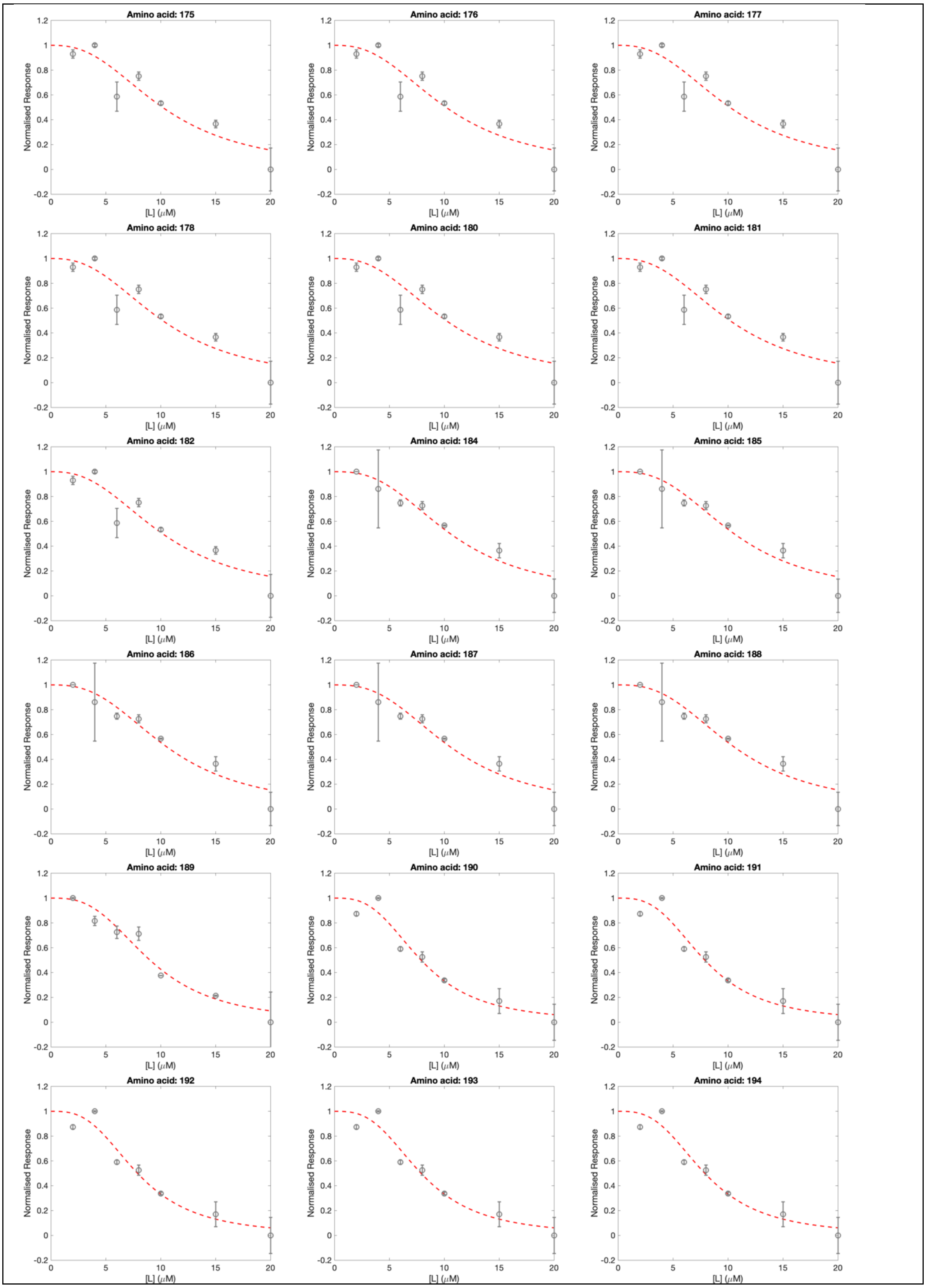
Amino acid: 175
Amino acid: 176
Amino acid: 177
Amino acid: 178
Amino acid: 180
Amino acid: 181
Amino acid: 182
Amino acid: 184
Amino acid: 185
Amino acid: 186
Amino acid: 187
Amino acid: 188
Amino acid: 189
Amino acid: 190
Amino acid: 191
Amino acid: 192
Amino acid: 193
Amino acid: 194
Normalised Response
[L] (μM)

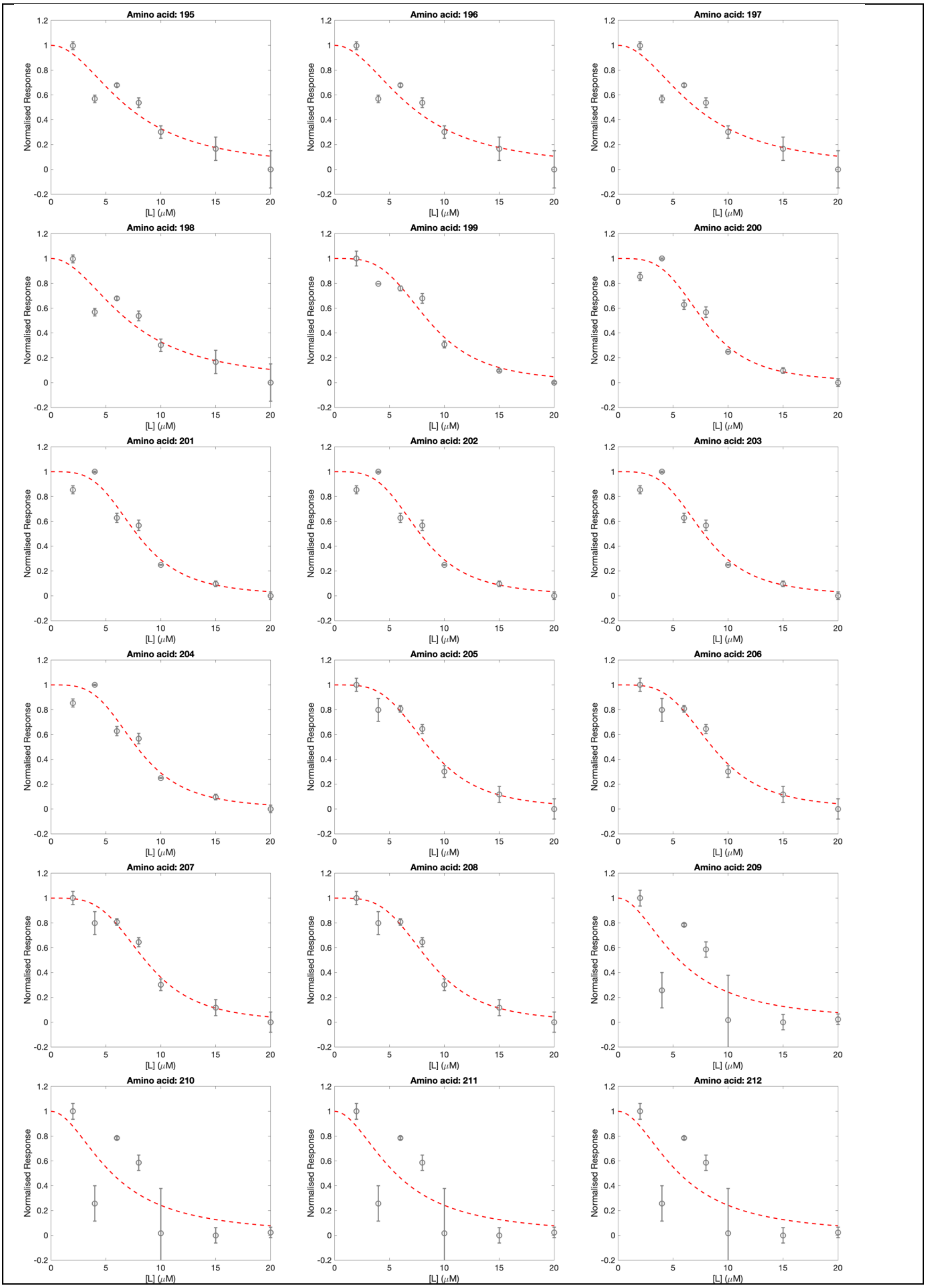
Amino acid: 195
Amino acid: 196
Amino acid: 197
Amino acid: 198
Amino acid: 199
Amino acid: 200
Amino acid: 201
Amino acid: 202
Amino acid: 203
Amino acid: 204
Amino acid: 205
Amino acid: 206
Amino acid: 207
Amino acid: 208
Amino acid: 209
Amino acid: 210
Amino acid: 211
Amino acid: 212
Normalised Response
[L] (μM)

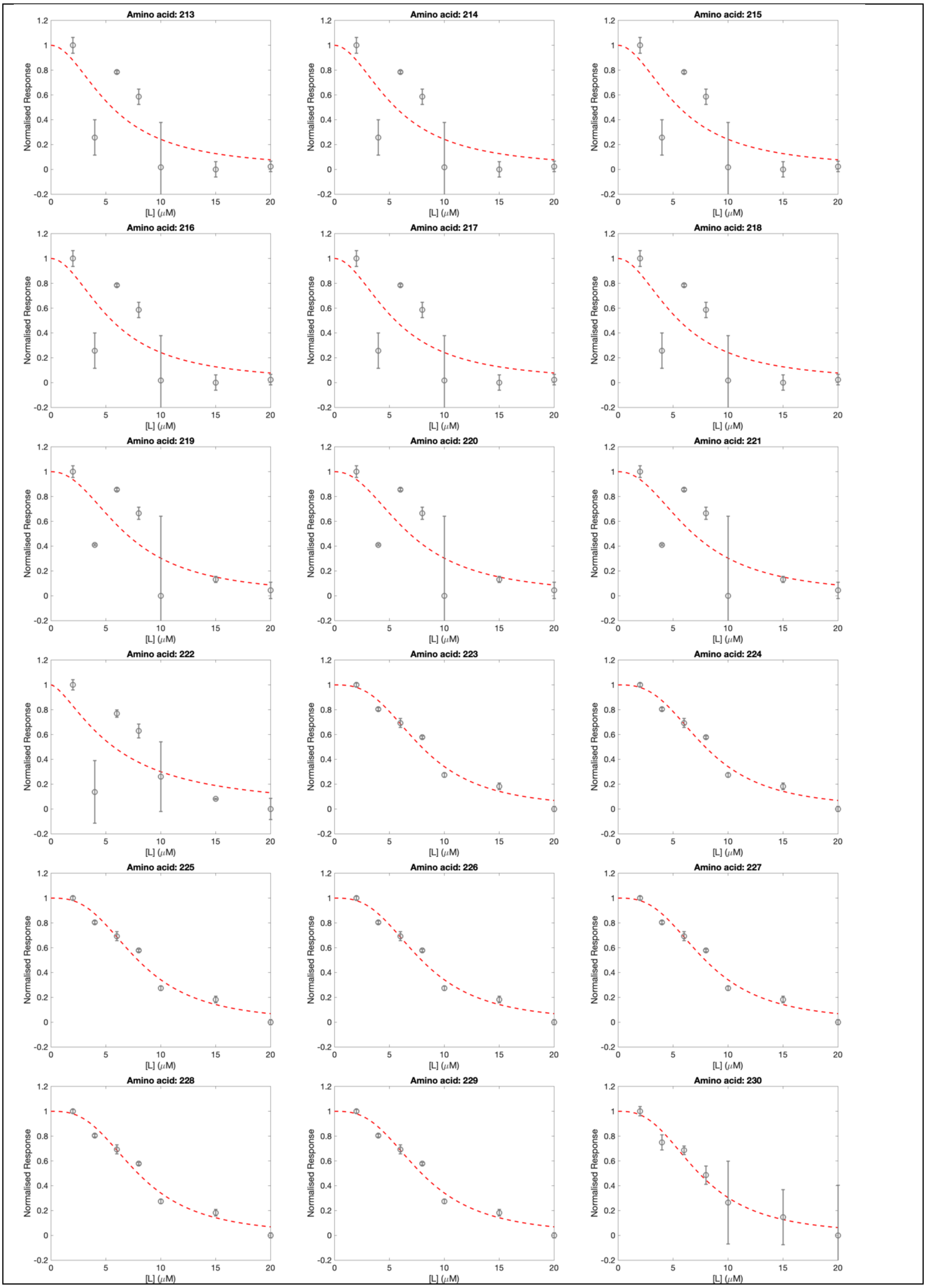
Amino acid: 213
Amino acid: 214
Amino acid: 215
Amino acid: 216
Amino acid: 217
Amino acid: 218
Amino acid: 219
Amino acid: 220
Amino acid: 221
Amino acid: 222
Amino acid: 223
Amino acid: 224
Amino acid: 225
Amino acid: 226
Amino acid: 227
Amino acid: 228
Amino acid: 229
Amino acid: 230
Normalised Response
[L] (μM)

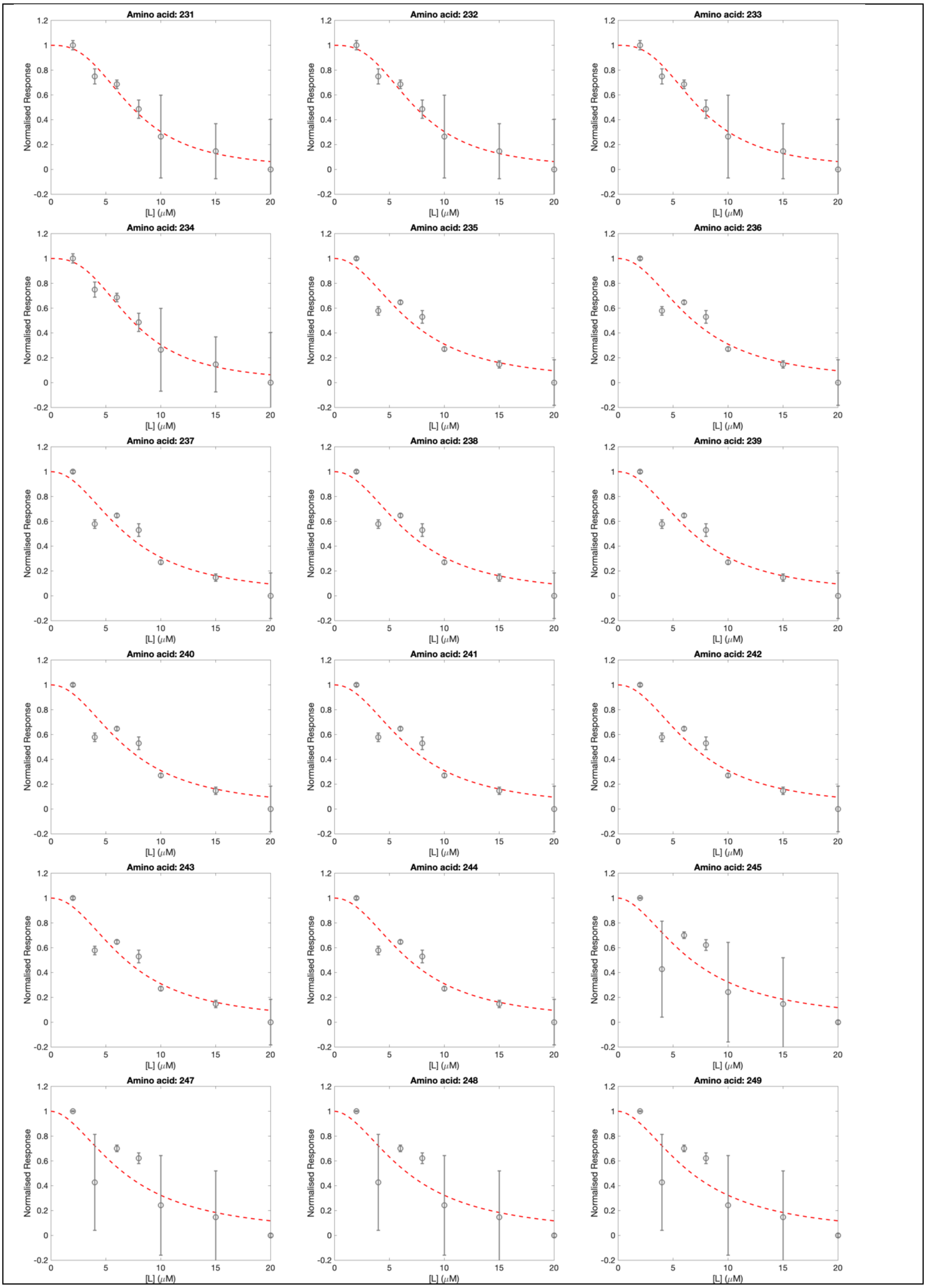
Amino acid: 231
Amino acid: 232
Amino acid: 233
Amino acid: 234
Amino acid: 235
Amino acid: 236
Amino acid: 237
Amino acid: 238
Amino acid: 239
Amino acid: 240
Amino acid: 241
Amino acid: 242
Amino acid: 243
Amino acid: 244
Amino acid: 245
Amino acid: 247
Amino acid: 248
Amino acid: 249
Normalised Response
[L] (μM)

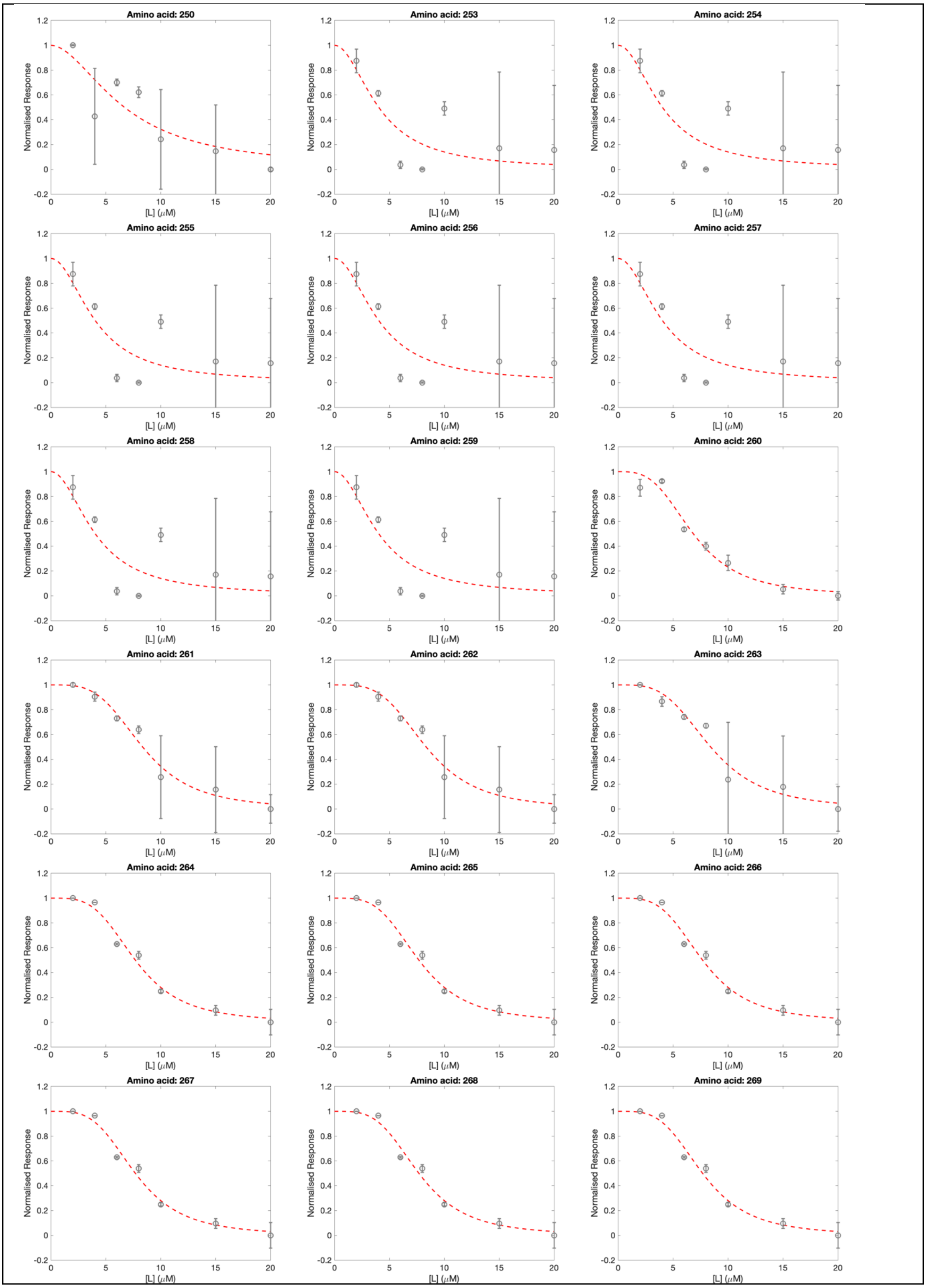
Amino acid: 250
Amino acid: 253
Amino acid: 254
Amino acid: 255
Amino acid: 256
Amino acid: 257
Amino acid: 258
Amino acid: 259
Amino acid: 260
Amino acid: 261
Amino acid: 262
Amino acid: 263
Amino acid: 264
Amino acid: 265
Amino acid: 266
Amino acid: 267
Amino acid: 268
Amino acid: 269
Normalised Response
[L] (μM)

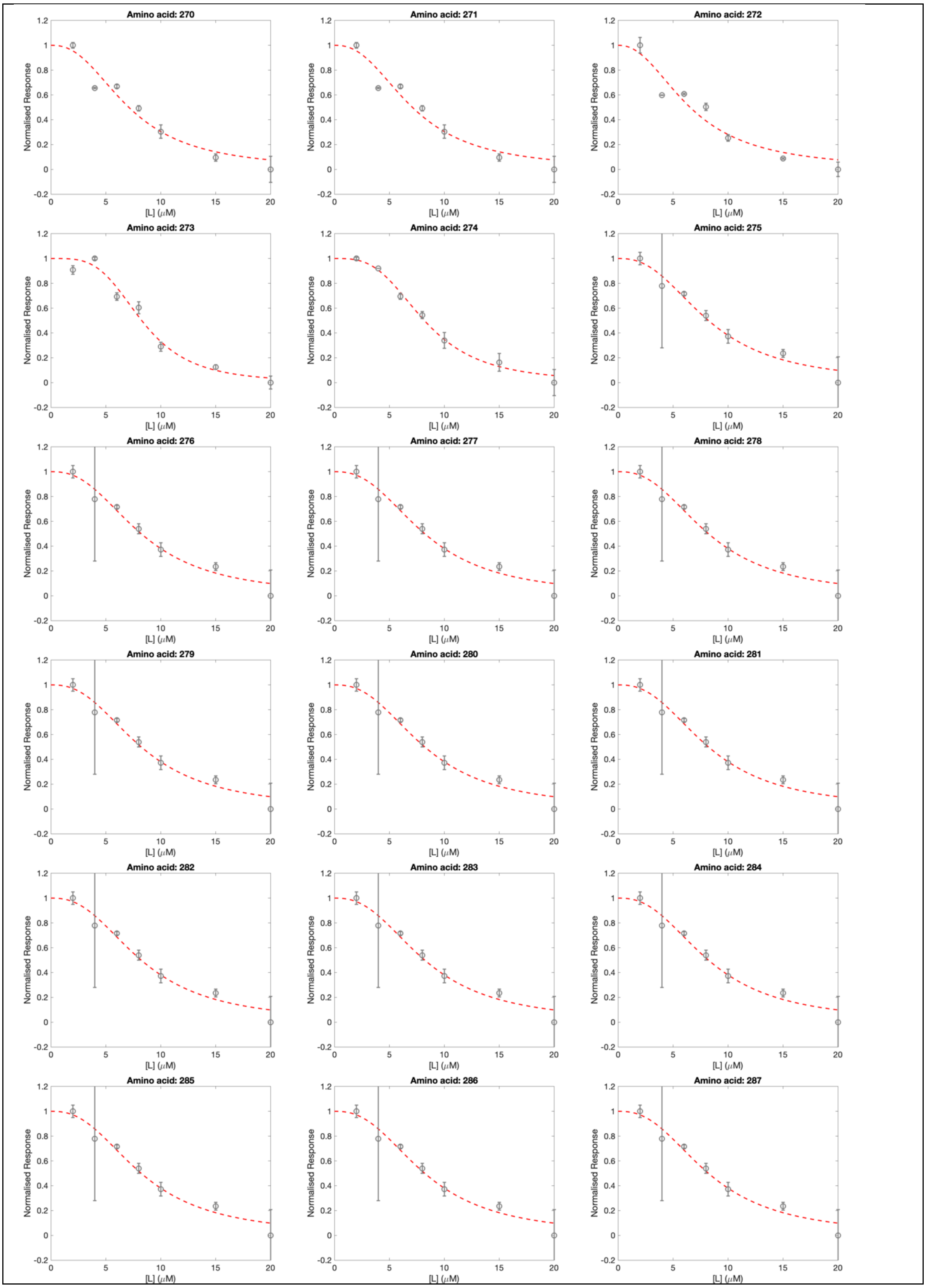
Amino acid: 270
Amino acid: 271
Amino acid: 272
Amino acid: 273
Amino acid: 274
Amino acid: 275
Amino acid: 276
Amino acid: 277
Amino acid: 278
Amino acid: 279
Amino acid: 280
Amino acid: 281
Amino acid: 282
Amino acid: 283
Amino acid: 284
Amino acid: 285
Amino acid: 286
Amino acid: 287
Normalised Response
[L] (μM)

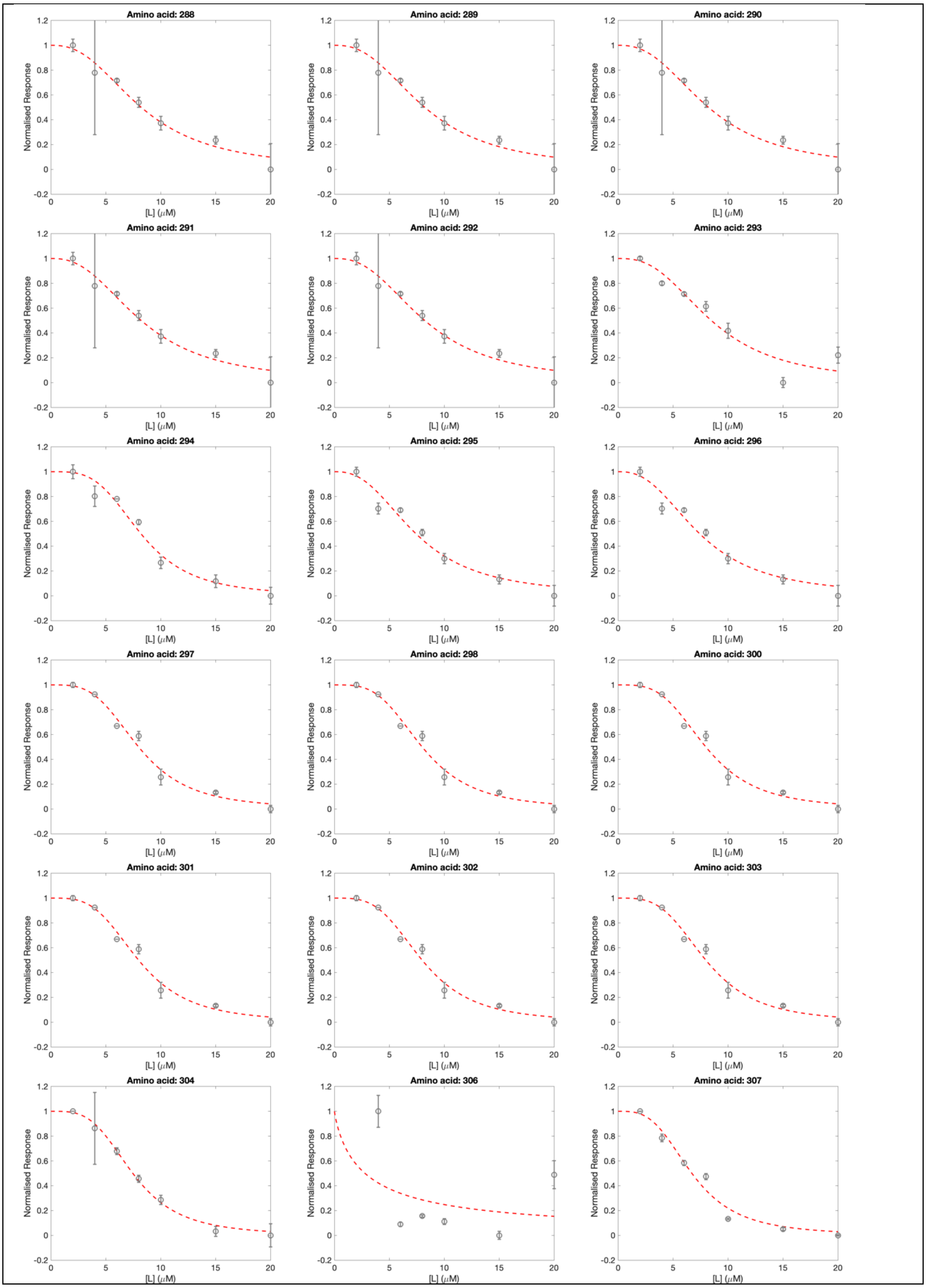
Amino acid: 288
Amino acid: 289
Amino acid: 290
Amino acid: 291
Amino acid: 292
Amino acid: 293
Amino acid: 294
Amino acid: 295
Amino acid: 296
Amino acid: 297
Amino acid: 298
Amino acid: 300
Amino acid: 301
Amino acid: 302
Amino acid: 303
Amino acid: 304
Amino acid: 306
Amino acid: 307
Normalised Response
[L] (μM)

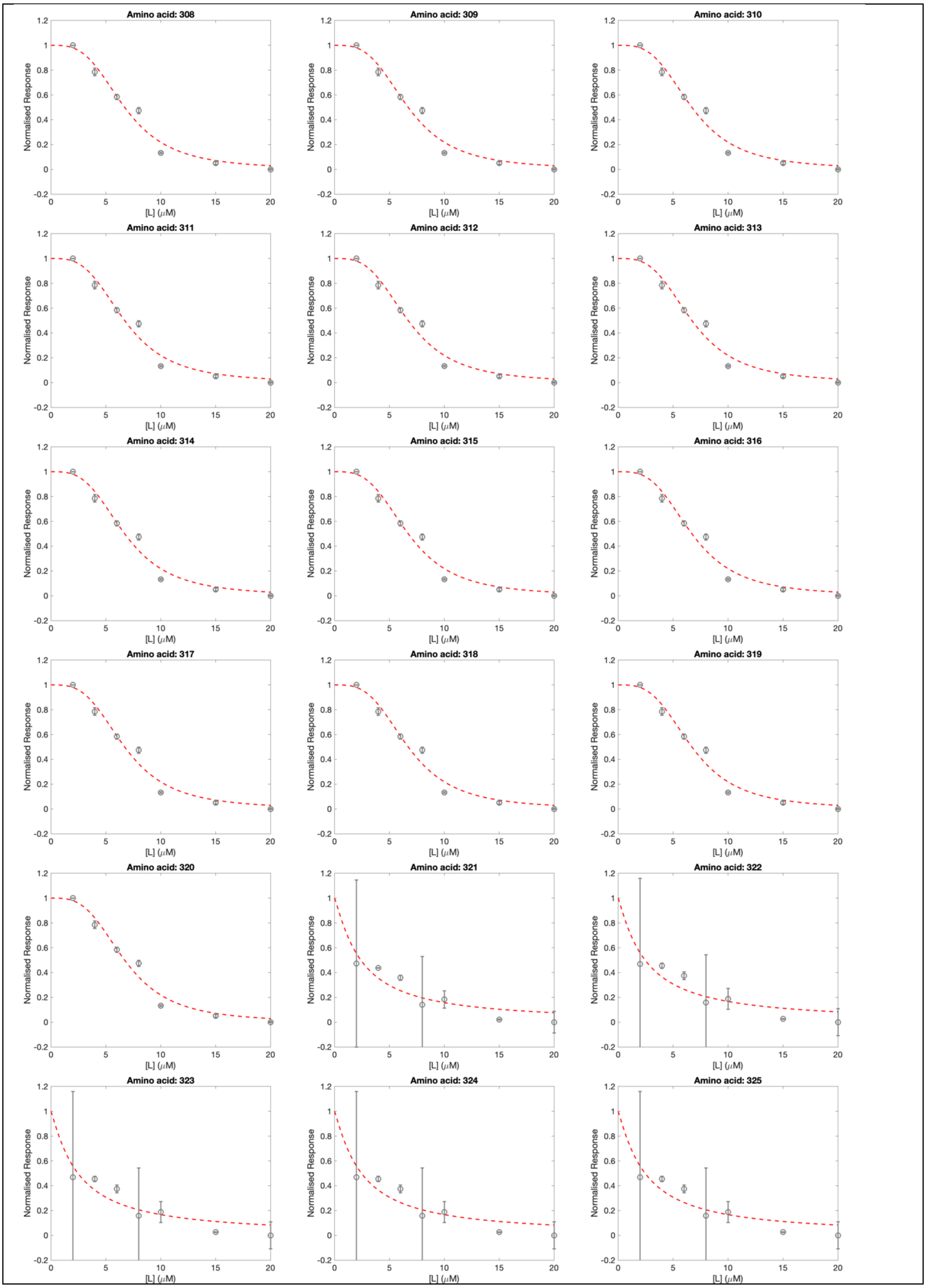
Amino acid: 308
Amino acid: 309
Amino acid: 310
Amino acid: 311
Amino acid: 312
Amino acid: 313
Amino acid: 314
Amino acid: 315
Amino acid: 316
Amino acid: 317
Amino acid: 318
Amino acid: 319
Amino acid: 320
Amino acid: 321
Amino acid: 322
Amino acid: 323
Amino acid: 324
Amino acid: 325
Normalised Response
[L] (μM)

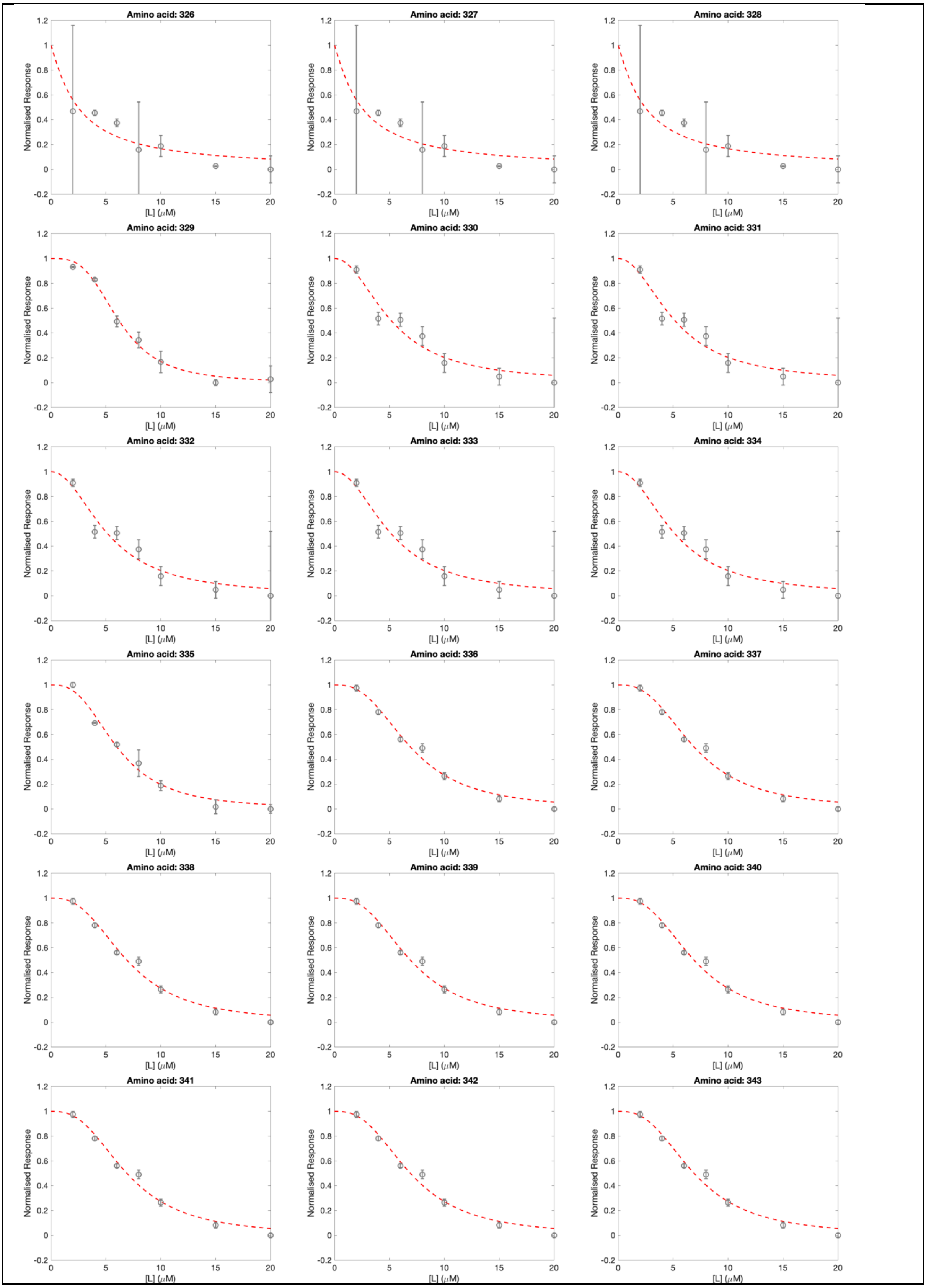
Amino acid: 326
Amino acid: 327
Amino acid: 328
Amino acid: 329
Amino acid: 330
Amino acid: 331
Amino acid: 332
Amino acid: 333
Amino acid: 334
Amino acid: 335
Amino acid: 336
Amino acid: 337
Amino acid: 338
Amino acid: 339
Amino acid: 340
Amino acid: 341
Amino acid: 342
Amino acid: 343
Normalised Response
[L] (μM)

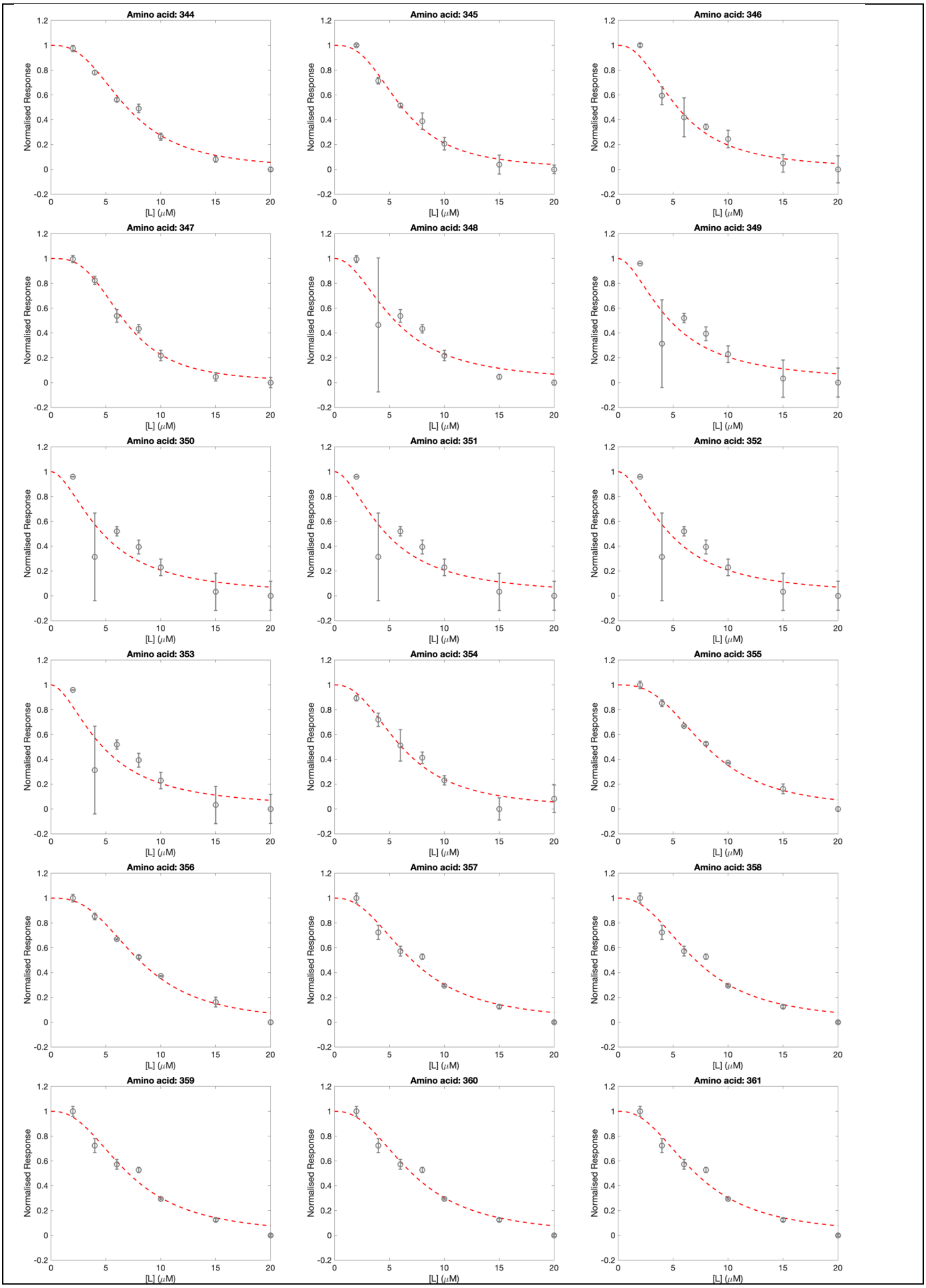
Amino acid: 344
Amino acid: 345
Amino acid: 346
Amino acid: 347
Amino acid: 348
Amino acid: 349
Amino acid: 350
Amino acid: 351
Amino acid: 352
Amino acid: 353
Amino acid: 354
Amino acid: 355
Amino acid: 356
Amino acid: 357
Amino acid: 358
Amino acid: 359
Amino acid: 360
Amino acid: 361
Normalised Response
[L] (μM)

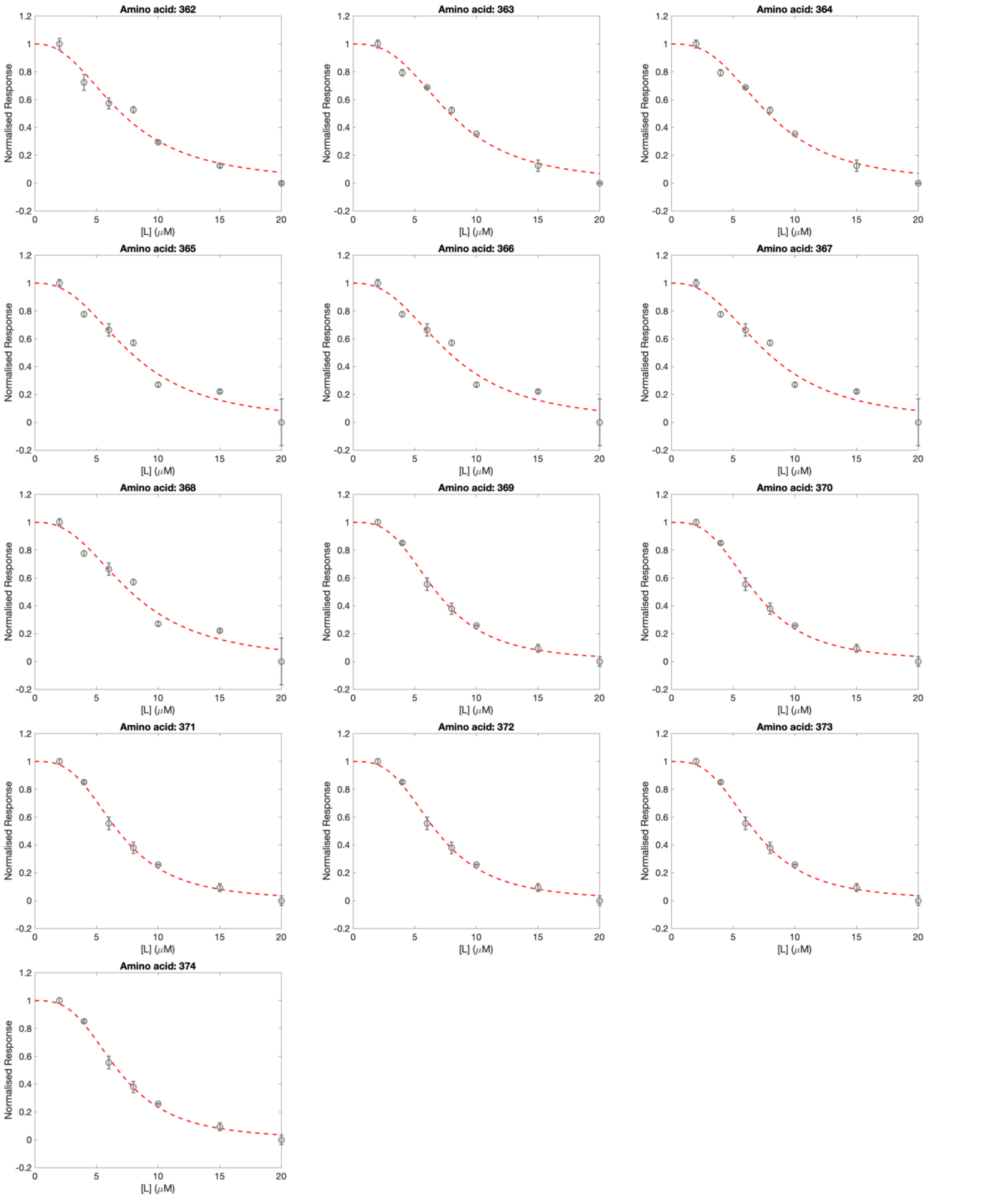

Supplementary figure 9. Normalized relative deuterium incorporation curves as a function of rapamycin concentration for all of the FKBP-cpNanoluc-FRB pepsin digest fragments (peptides) identified in supplementary figure 6 under conditions stated in Table S2. HDX measurements for FKBP-cpNanoluc-FRB peptides at different deuterium labelling times across three orders of magnitude (0, 30, 300 and 3000 s) were summed and linearly weighted by maximal uptake using the heatmap function in DynamX (Waters) to obtain total deuterium uptake resolved per amino acid. These were then normalized per amino acid to the maximum uptake observed over the rapamycin titration. Nonlinear regression to

a normalized Hill dose-response curve (SI equation 2) was done with lsqcurvefit() in Matlab (Mathworks). Data (open circles) were the mean of all assigned charge states for n=2 independent experiments with different protein stocks, summed over three time points. Error bars (+/- 1 s.d.). Fit to Hill equation (red dashed line).

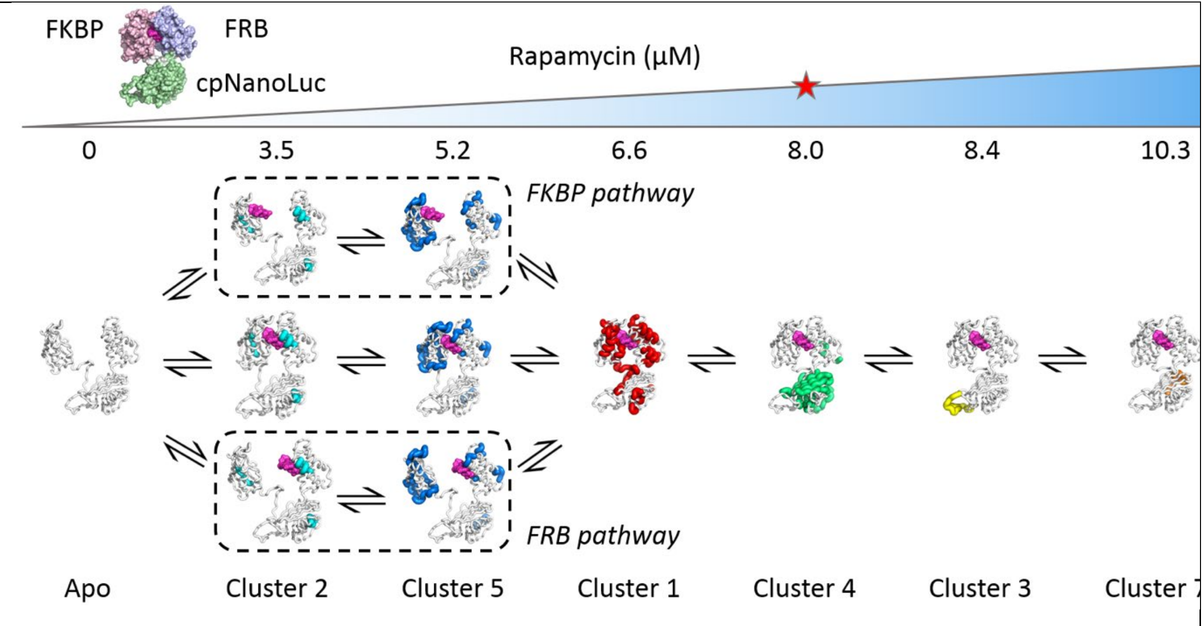

Supplementary figure 10. Clusters of amino acids with rapamycin dose-response, as determined by hydrogen-exchange, mapped onto structural models of FKBP-cpNanoLuc-FRB to reveal the structural equilibrium pathway of activation. Rapamycin (purple) binds to the FKBP (pink) and FRB (blue) domains and stabilizes a 'closed' form of the central cpNanoLuc (green) domain in which the β-10 strand has intercalated into the β-sheet. The centroid of each cluster has its midpoint at the given concentrations. Parallel pathways exist for ligand binding, though the central pathway (simultaneous FKBP/FRB binding to rapamycin) is improbable. Few structural changes beyond 1:1 stoichiometry of binding (8 μM) where the biosensor is 96% bound to rapamycin.

Supplementary Table 1 Sequences of the open reading frames encoding recombinant proteins used in this study

Construct Name	Sequence
CaM-NanoLuc-FKBP	GSDNMVFTLEDFVGDWRQTAGYNLDQVLEQGGVSSLFQNLGVSVTPIQRIVLSGE NGLKIDIHVIIPYEGLSGDQMGQIEKIFKVVYPVDDHHFKVILHYGTLVIDGVTPNMI

(Rapmycin/FK506 sensor component 1)	DYFGRPYEGIAVFDGKKITVTGTLWNGNKIIDERLINPDGSLLFRVTINGSGGTEEQIAEFKEAFSLFDKDGDGTITTKELGTVMRSLGQNPTEAELQDMINEVDADGNGTIDFPEFLTMMARKMKDTDSEEEIREAFRVFDKDGNGYISAAELRHVMTNLGEKLTDEEVDEMIREADIDGDGQVNYEEFVQMMTAGGSGGVTGWRLCERILAGGSGSGGGVQVETISPGDGRTFPKRGQTCVVHYTGMLEDGKKFDSSRDRNKPFKFMLGKQEVIRGWEEGVAQMSVGQRAKLTISPDYAYGATGHPGIIPPHATLVFDVELLKLEKLAAALEHHHHHH
FRB-CaM-BP (Rapmycin sensor component 2)	AHHHHHHSSGTRVAILWHEMWHEGLEEASRLYFGERNVKGMFEVLEPLHAMMERGPQTLKETSFNQAYGRDLMEAQEWCRKYMKSGNVKDLTQAWDLYYHVFRRISGGSGGSGSGSGGSGGKRRWKKNFIAVASASA
Calcineurin A-Calcineurin B- CaM-BP (FK506 sensor component 2)	HHHHHHSSGTSEPKAIDPKLSTTDRVVKAVPFPPSHRLTAKEVFDNDGKPRVDILKAHLMKEGRLEESVALRIITEGASILRQEKNLLDIDAPVTVCGDIHGQFFDLMKLFEVGGSPANTRYLFLGDYVDRGYFSIECVLYLWALKILYPKTLFLLRGNHECRHLTEYFTFKQECKIKYSERVYDACMDAFDCLPLAALMNQQFLCVHGGLSPEINTLDDIRKLDRFKEPPAYGPMCDILWSDPLEDFGNEKTQEHFTHNTVRGCSYFYSYPAVCEFLQHNNLLSILRAHEAQDAGYRMYRKSQTTGFPSLITIFSAPNYLDVYNNKAAVLKYENNVMNIRQFNCSPHPYWLPNFMDVFTWSLPFVGEKVTEMLVNVLNICSDDELGSEEDGSGSGSGGGNEASYPLEMCSHFDADEIKRLGKRFKKLDLDNSGSLSVEEFMSLPELQQNPLVQRVIDIFDTDGNGEVDFKEFIEGVSQFSVKGDKEQKLRFAFRIYDMDKDGYISNGELFQVLKMMVGNNLKDTQLQQIVDKTIINADKDGDGRISFEEFCAVVGGLDIHKKMVVDVGGSGGSGSGSGGSGGKRRWKKNFIAVASASA
FKBP-cpNanoLuc-FRB (Rapmycin sensor)	DHHHHHHGVQVETISPGDGRTFPKRGQTCVVHYTGMLEDGKKFDSSRDRNKPFKFMLGKQEVIRGWEEGVAQMSVGQRAKLTISPDYAYGATGHPGIIPPHATLVFDVELLKLEGGSGGSGGVTGWRLCERILAGGSGSGSGGSGSGGSDNMVFTLEDFVGDWRQTAGYNLDQVLEQGGVSSLFQNLGVSVTPIQRIVLSGENGLKIDIHVIIPYEGLSGDQMGQIEKIFKVVYPVDDHHFKVILHYGTLVIDGVTPNMIDYFGRPYEGIAVFDGKKITVTGTLWNGNKIIDERLINPDGSLLFRVTINGSGSGSGGLWHEMWHEGLEEASRLYFGERNVKGMFEVLEPLHAMMERGPQTLKETSFNQAYGRDLMEAQEWCRKYMKSGNVKDLTQAWDLYYHVFRRIS
Calcineurin A-Calcineurin B -cpNanoLuc-FKBP (FK506 sensor)	DHHHHHHSSGTSEPKAIDPKLSTTDRVVKAVPFPPSHRLTAKEVFDNDGKPRVDILKAHLMKEGRLEESVALRIITEGASILRQEKNLLDIDAPVTVCGDIHGQFFDLMKLFEVGGSPANTRYLFLGDYVDRGYFSIECVLYLWALKILYPKTLFLLRGNHECRHLTEYFTFKQECKIKYSERVYDACMDAFDCLPLAALMNQQFLCVHGGLSPEINTLDDIRKLDRFKEPPAYGPMCDILWSDPLEDFGNEKTQEHFTHNTVRGCSYFYSYPAVCEFLQHNNLLSILRAHEAQDAGYRMYRKSQTTGFPSLITIFSAPNYLDVYNNKAAVLKYENNVMNIRQFNCSPHPYWLPNFMDVFTWSLPFVGEKVTEMLVNVLNICSDDELGSEEDGSGSGSGGGNEASYPLEMCSHFDADEIKRLGKRFKKLDLDNSGSLSVEEFMSLPELQQNPLVQRVIDIFDTDGNGEVDFKEFIEGVSQFSVKGDKEQKLRFAFRIYDMDKDGYISNGELFQVLKMMVGNNLKDTQLQQIVDKTIINADKDGDGRISFEEFCAVVGGLDIHKKMVVDVGGSGSGSGGVTGWRLCERILAGGSGSGSGGSGSGGSDNMVFTLEDFVGDWRQTAGYNLDQVLEQGGVSSLFQNLGVSVTPIQRIVLSGENGLKIDIHVIIPYEGLSGDQMGQIEKIFKVVYPVDDHHFKVILHYGTLVIDGVTPNMIDYFGRPYEGIAVFDGKKITVTGTLWNGNKIIDERLINPDGSLLFRVTINGSGSSGSGGGVQVETISPGDGRTFPKRGQTCVVHYTGMLEDGKKFDSSRDRNKPFKFMLGKQEVIRGWEEGVAQMSVGQRAKLTISPDYAYGATGHPGIIPPHATLVFDVELLKLE
VHH1 -cpNanoLuc-VHH2 (amylase sensor)	DHHHHHHDTTVSEPAPSCVTLYQSWRYSQADNGCAETVTVKVVYEDDTEGLCYAVAPGQITTVGDGYIGSHGHARYLARCLGGSGGSGGVTGWRLCERILAGGSGSGSGSGGSGSGGSDNMVFTLEDFVGDWRQTAGYNLDQVLEQGGVSSLFQNLGVSVTPIQRIVLSGENGLKIDIHVIIPYEGLSGDQMGQIEKIFKVVYPVDDHHFKVILHYGTLVIDGVTPNMIDYFGRPYEGIAVFDGKKITVTGTLWNGNKIIDERLINPDGSLLFRVTINGSG

	SGSGGQVQLVESGGGTVPAGGSLRLSCAASGNTLCTYDMSWYRRAPGKGRDFVS<br>GIDNDGTTTYVDSVAGRFTISQGNAKNTAYLQMDSLKPDDTAMYYCKPSLRYGLPG<br>CPIIPWGQGTQVTVSS
VHH1 -cpNanoLuc-VHH2 (HSA sensor)	QVQLQESGGGLVQAGGSLRLSCAASGYISDAYYMGWYRQAPGKEREFVATITHGT<br>NTYYADSVKGRFTISRDNAKNTVYLQMNSLKPEDTAVYYCAVLETRSYSFRYWGQG<br>TQVTVSSGGSGSGSSDSGSGGSDGSSGSGSSGSDSSGSGDGSGGGVTGWRLCERIL<br>AGGSGSGSGSGGSGSGGSDNMVFTLEDFVGDWRQTAGYNLDQVLEQGGVSSLF<br>QNLGVSVTPIQRIVLSGENGLKIDIHVIIPYEGLSGDQMGQIEKIFKVVYPVDDHHFK<br>VILHYGTLVIDGVTPNMIDYFGRPYEGIAVFDGKKITVTGTLWNGNKIIDERLINPDG<br>SLLFRVTINGGGSSGGSSDSGGGSDSGSGSDGSGSGSGGSGGGPGGGAVDANSLA<br>EAKVLANRELDKYGVSDFYKRLINKAKTVEGVEALKLHILAALPKLAAALEHHHHHH
FKBP-cpNanoLuc—eGFP-FRB (Rapamycin sensor)	DHHHHHHGVQVETISPGDGRTFPKRGQTCVVHYTGMLEDGKKFDSSRDRNKPFKF<br>MLGKQEVIRGWEEGVAQMSVGQRAKLTISPDYAYGATGHPGIIPPHATLVFDVEL<br>LKLEGGSGGSGGVTGWRLCERILAGGSGSGGQLVSKGEELFTGVVPILVELDGDV<br>NGHKFSVSGEGEGDATYGKLTLKFICTTGKLPVPWPTLVTTLTYGVQCFSRYPDH<br>MKQHDFFKSAMPEGYVQERTIFFKDDGNYKTRAEVKFEGDTLVNRIELKGIDFKE<br>DGNILGHKLEYNYNSHNVYIMADKQKNGIKVNFKIRHNIEDGSVQLADHYQQNTP<br>IGDGPVLLPDNHYLSTQSALSKDPNEKRDHMVLLEFVTAAGITLGMDELYKGGGS<br>GGSDNMVFTLEDFVGDWRQTAGYNLDQVLEQGGVSSLFQNLGVSVTPIQRIVLSG<br>ENGLKIDIHVIIPYEGLSGDQMGQIEKIFKVVYPVDDHHFKVILHYGTLVIDGVT<br>PNMIDYFGRPYEGIAVFDGKKITVTGTLWNGNKIIDERLINPDGSLLFRVTINGS<br>GSGSGGLWHEMWHEGLEEASRLYFGERNVKGMFEVLEPLHAMMERGPQTLKETSF<br>NQAYGRDLMEAQEWCRKYMKSGNVKDLTQAWDLYYHVFRRIS
FKBP-cpLumiLuc-FRB (Rapamycin sensor)	DHHHHHHGVQVETISPGDGRTFPKRGQTCVVHYTGMLEDGKKFDSSRDRNKPFKF<br>MLGKQEVIRGWEEGVAQMSVGQRAKLTISPDYAYGATGHPGIIPPHATLVFDVEL<br>LKLEGGSGGSGGVTGWRLHERILAGGSGSGSGSGGSGSGGSDNMVFTLGDFVGDW<br>RQTAGYNQAQVLEQGGLTSLFQNLGVSVTPIQRIVLSGENGLKIDIHVIIPYEGL<br>SCDQMAQIEKIFKVVYPVDDHHFKAILHYGTLVIDGVTPNMIDYFGQPYEGIAKF<br>DGKKITVTGTLWNGNTIIDERLINPDGSLLFRVTINGSGSGSGGLWHEMWHEGLE<br>EASRLYFGERNVKGMFEVLEPLHAMMERGPQTLKETSFNQAYGRDLMEAQEWCRK<br>YMKSGNVKDLTQAWDLYYHVFRRIS
FKBP-cpLumiLuc-FRB-mScarlet (Rapamycin sensor)	DHHHHHHGVQVETISPGDGRTFPKRGQTCVVHYTGMLEDGKKFDSSRDRNKPFKF<br>MLGKQEVIRGWEEGVAQMSVGQRAKLTISPDYAYGATGHPGIIPPHATLVFDVEL<br>LKLEGGSGGSGGVTGWRLHERILAGGSGSGSGSGGSGSGGSDNMVFTLGDFVGDW<br>RQTAGYNQAQVLEQGGLTSLFQNLGVSVTPIQRIVLSGENGLKIDIHVIIPYEGL<br>SCDQMAQIEKIFKVVYPVDDHHFKAILHYGTLVIDGVTPNMIDYFGQPYEGIAKF<br>DGKKITVTGTLWNGNTIIDERLINPDGSLLFRVTINGSGSGSGGLWHEMWHEGLE<br>EASRLYFGERNVKGMFEVLEPLHAMMERGPQTLKETSFNQAYGRDLMEAQEWCRK<br>YMKSGNVKDLTQAWDLYYHVFRRISGGMVSKGEAVIKEFMRFKVHMEGSMNGHEF<br>EIEGEGEGRPYEGTQTAKLKVTKGGPLPFSWDILSPQFMYGSRAFIKHPADIPDY<br>YKQSFPEGFKWERVMNFEDGGAVTVTQDTSLEDGTLIYKVKLRGTNFPPDGPVMQ<br>KKTMGWEASTERLYPEDGVLKGDIKMALRLKDGGRYLADFKTTYKAKKPVQMPGA<br>YNVDRKLDITSHNEDYTVVEQYERSEGRHSTG
FKBP-cpLumiLuc-mScarlet-FRB (Rapamycin sensor)	DHHHHHHGVQVETISPGDGRTFPKRGQTCVVHYTGMLEDGKKFDSSRDRNKPFKF<br>MLGKQEVIRGWEEGVAQMSVGQRAKLTISPDYAYGATGHPGIIPPHATLVFDVEL<br>LKLEGGSGGSGGVTGWRLHERILAGGSGSGSGMVSKGEAVIKEFMRFKVHMEGSM<br>NGHEFEIEGEGEGRPYEGTQTAKLKVTKGGPLPFSWDILSPQFMYGSRAFIKHPA<br>DIPDYYKQSFPEGFKWERVMNFEDGGAVTVTQDTSLEDGTLIYKVKLRGTNFPPD<br>GPVMQKKTMGWEASTERLYPEDGVLKGDIKMALRLKDGGRYLADFKTTYKAKKPV<br>QMPGAYNVDRKLDITSHNEDYTVVEQYERSEGRHSTGSGGSGSGGSDNMVFTLGD<br>FVGDWRQTAGYNQAQVLEQGGLTSLFQNLGVSVTPIQRIVLSGENGLKIDIHVII<br>PYEGLSCDQMAQIEKIFKVVYPVDDHHFKAILHYGTLVIDGVTPNMIDYFGQPYE<br>GIAKFDGKKITVTGTLWNGNTIIDERLINPDGSLLFRVTINGSGSGSGGLWHEMW<br>HEGLEEASRLYFGERNVKGMFEVLEPLHAMMERGPQTLKETSFNQAYGRDLMEAQ<br>EWCRKYMKSGNVKDLTQAWDLYYHVFRRIS

VHH1 -cpLumiLuc-VHH2 (amylase sensor)	DHHHHHHDTTVSEPAPSCVTLYQSWRYSQADNGCAETVTVKVVYEDDTEGLCYAVAPGQITTVGDGYIGSHGHARYLARCLGGSGGSGGVTGWRLHERILAGGSGSGSGSGGSGSGGSDNMVFTLGDFVGDWRQTAGYNQAQVLEQGGLTSLFQNLGVSVTPIQRIVLSGENGLKIDIHVIIPYEGLSCDQMAQIEKIFKVVYPVDDHHFKAILHYGTLVIDGVTPNMIDYFGQPYEGIAKFDGKKITVTGTLWNGNTIIDERLINPDGSLLFRVTINGSGSGSGGQVQLVESGGGTVPAGGSLRLSCAASGNTLCTYDMSWYRRAPGKGRDFVSGIDNDGTTTYVDSVAGRFTISQGNAKNTAYLQMDSLKPDDTAMYYCKPSLRYGLPGCPIIPWGQGTQVTVSS
FKBP-wtNanoLuc-FRB	DHHHHHHGVQVETISPGDGRTFPKRGQTCVVHYTGMLEDGKKFDSSRDRNKPFKFMLGKQEVIRGWEEGVAQMSVGQRAKLTISPDYAYGATGHPGIIPPHATLVFDVELLKLEGGSGGSGGSDNMVFTLEDFVGDWRQTAGYNLDQVLEQGGVSSLFQNLGVSVTPIQRIVLSGENGLKIDIHVIIPYEGLSGDQMGQIEKIFKVVYPVDDHHFKVILHYGTLVIDGVTPNMIDYFGRPYEGIAVFDGKKITVTGTLWNGNKIIDERLINPDGSLLFRVTINGVTGWRLCERILAGSGSGSGGLWHEMWHEGLEEASRLYFGERNVKGMFEVLEPLHAMMERGPQTLKETSFNQAYGRDLMEAQEWCRKYMKSGNVKDLTQAWDLYYHVFRRIS
cpNanoLuc	DHHHHHHVTGWRLCERILAGGSGSGSGGSGSGGSDNMVFTLEDFVGDWRQTAGYNLDQVLEQGGVSSLFQNLGVSVTPIQRIVLSGENGLKIDIHVIIPYEGLSGDQMGQIEKIFKVVYPVDDHHFKVILHYGTLVIDGVTPNMIDYFGRPYEGIAVFDGKKITVTGTLWNGNKIIDERLINPDGSLLFRVTIN
GS linker-cpNanoLuc-GS linker	DHHHHHHGSGSGSGSGGSGGSGSSGSGSGGSSGGSGGVTGWRLCERILAGGSGSGSGSGGSGSGGSDNMVFTLEDFVGDWRQTAGYNLDQVLEQGGVSSLFQNLGVSVTPIQRIVLSGENGLKIDIHVIIPYEGLSGDQMGQIEKIFKVVYPVDDHHFKVILHYGTLVIDGVTPNMIDYFGRPYEGIAVFDGKKITVTGTLWNGNKIIDERLINPDGSLLFRVTINGSGSGGSGGSGSSGSGSGSSGGSGSGGSGG

Supplementary figure 2 Summary of mass spectrometry experimental details

Dataset	FKBP-cpNanoluc-FRB (8 μM), FKBP-cpNanoluc-FRB + Rapamycin (2 μM, 4 μM, 6 μM, 8 μM, 10 μM, 15 μM, 20 μM)
HDX reaction details	20 mM Tris buffer containing 100 mM NaCl pH 7.2; 20 mM Tris buffer containing 100 mM NaCl pD 7.2
HDX time course	30, 300, 3000 s
HDX controls	0 s
Number of peptides	107
Sequence coverage	97.3 %
Redundancy	3.53
Replicates	Technical replicates -2
Significant differences in HDX	Data were fitted as a function of ligand and clustered accordingly – no significant difference in HDX was measured directly.
