# Supplementary material for: Engineering and exploiting synthetic allostery of NanoLuc luciferase
Source: Nat Commun. 2022 Feb 10;13:789. doi: 10.1038/s41467-022-28425-2 (PMC8831504; doi:10.1038/s41467-022-28425-2)
Supplement: Supplementary file 2 — Reporting Summary [file 41467_2022_28425_MOESM2_ESM.pdf]

nature research

Corresponding author(s): Kirill Alexandrov

Last updated by author(s): Jan 23, 2022

Reporting Summary

Nature Research wishes to improve the reproducibility of the work that we publish. This form provides structure for consistency and transparency in reporting. For further information on Nature Research policies, see our Editorial Policies and the Editorial Policy Checklist.

Statistics

For all statistical analyses, confirm that the following items are present in the figure legend, table legend, main text, or Methods section.

n/a	Confirmed	
☐	☒	The exact sample size (*n*) for each experimental group/condition, given as a discrete number and unit of measurement
☐	☒	A statement on whether measurements were taken from distinct samples or whether the same sample was measured repeatedly
☒	☐	The statistical test(s) used AND whether they are one- or two-sided<br>*Only common tests should be described solely by name; describe more complex techniques in the Methods section.*
☐	☒	A description of all covariates tested
☒	☐	A description of any assumptions or corrections, such as tests of normality and adjustment for multiple comparisons
☐	☒	A full description of the statistical parameters including central tendency (e.g. means) or other basic estimates (e.g. regression coefficient) AND variation (e.g. standard deviation) or associated estimates of uncertainty (e.g. confidence intervals)
☒	☐	For null hypothesis testing, the test statistic (e.g. *F*, *t*, *r*) with confidence intervals, effect sizes, degrees of freedom and *P* value noted<br>*Give P values as exact values whenever suitable.*
☒	☐	For Bayesian analysis, information on the choice of priors and Markov chain Monte Carlo settings
☒	☐	For hierarchical and complex designs, identification of the appropriate level for tests and full reporting of outcomes
☒	☐	Estimates of effect sizes (e.g. Cohen's *d*, Pearson's *r*), indicating how they were calculated

Our web collection on statistics for biologists contains articles on many of the points above.

Software and code

Policy information about availability of computer code

Data collection	Masslynx v4.1 SCN957 (Waters)
Data analysis	Protein Lynx Global Server v3.0.3 (Waters); DynamX v3.0 (Waters); Matlab R2019a (Mathworks); Pymol 2.3.5 (Schrodinger); Prism v8 (Graphpad), Grafit 5.0, MassLynx v4.1 SCN 957 (Waters)

For manuscripts utilizing custom algorithms or software that are central to the research but not yet described in published literature, software must be made available to editors and reviewers. We strongly encourage code deposition in a community repository (e.g. GitHub). See the Nature Research guidelines for submitting code & software for further information.

Data

Policy information about availability of data

All manuscripts must include a data availability statement. This statement should provide the following information, where applicable:

- Accession codes, unique identifiers, or web links for publicly available datasets
- A list of figures that have associated raw data
- A description of any restrictions on data availability

Hydrogen exchange mass spectrometry data is supplied in HydrogenExchangeData.xlsx. Source data for Figure 4 is provided in the source data file. Summaries of these are provided in Supplementary Figure 9. All mass spectrometry raw data files are available via ProteomeXchange with identifier PXD031169

Field-specific reporting

Please select the one below that is the best fit for your research. If you are not sure, read the appropriate sections before making your selection.

- [x] Life sciences
- [ ] Behavioural & social sciences
- [ ] Ecological, evolutionary & environmental sciences

For a reference copy of the document with all sections, see nature.com/documents/nr-reporting-summary-flat.pdf

Life sciences study design

All studies must disclose on these points even when the disclosure is negative.

Sample size	the sample size applies to the number of the concentrations of the cognate ligand tested for each individual biosensor. This was determined experimental to achieve at least 5 data points in the area of biosensors linear response response . The majority of titrations had at least 10 data points with intention of having at lest three data points distributed in the kink of the curve ensuring accurate data fit.
Data exclusions	no data was excluded
Replication	the replicates confirmed the observed effects, experiments were performed in parallel triplicates
Randomization	randomization was not relevant to this study
Blinding	Blinding was performed for the biomarker quantification in patient's samples . Here the samples received form the pathology lab did not have any identification information and were independently analyzed using developed analytic assay. The reference data was unblinded after the quantification results were obtained and used to generate the correlation plot presented in figure 3A,B

Reporting for specific materials, systems and methods

We require information from authors about some types of materials, experimental systems and methods used in many studies. Here, indicate whether each material, system or method listed is relevant to your study. If you are not sure if a list item applies to your research, read the appropriate section before selecting a response.

Materials & experimental systems

n/a	Involved in the study
x	☐ Antibodies
x	☐ Eukaryotic cell lines
x	☐ Palaeontology and archaeology
x	☐ Animals and other organisms
x	☐ Human research participants
x	☐ Clinical data
x	☐ Dual use research of concern

Methods

n/a	Involved in the study
x	☐ ChIP-seq
x	☐ Flow cytometry
x	☐ MRI-based neuroimaging
