# Supplementary material for: Engineering and exploiting synthetic allostery of NanoLuc luciferase
Source: Nat Commun. 2022 Feb 10;13:789. doi: 10.1038/s41467-022-28425-2 (PMC8831504; doi:10.1038/s41467-022-28425-2)
Supplement: Supplementary file 4 — Description of Additional Supplementary Files [file 41467_2022_28425_MOESM4_ESM.pdf]

Title: Supplementary data 1:
Description: Excel file with three sheets containing state data export from DynamX
